# Plantar pressures are elevated in people with longstanding diabetes-related foot ulcers during follow-up

**DOI:** 10.1371/journal.pone.0181916

**Published:** 2017-08-31

**Authors:** Malindu E. Fernando, Robert G. Crowther, Peter A. Lazzarini, Saiumaeswar Yogakanthi, Kunwarjit S. Sangla, Petra Buttner, Rhondda Jones, Jonathan Golledge

**Affiliations:** 1 Vascular Biology Unit, Queensland Research Centre for Peripheral Vascular Disease, College of Medicine and Dentistry, James Cook University, Townsville, Australia; 2 School of Clinical Sciences, Queensland University of Technology, Brisbane, Australia; 3 Movement Analysis Laboratory, Sports and Exercise Science, James Cook University, Townsville, Australia; 4 Podiatry Service, Kirwan Community Health Campus, Townsville, Queensland, Australia; 5 School of Health Sciences, University of South Australia, Adelaide, South Australia, Australia; 6 Allied Health Research Collaborative, Metro North Hospital & Health Service, Queensland Health, Brisbane, Australia; 7 Faculty of Medicine, Nursing and Health Science, Monash University, Melbourne, Australia; 8 Department of Diabetes and Endocrinology, The Townsville Hospital, Townsville, Queensland, Australia; 9 Centre for Chronic Disease Prevention, James Cook University, Cairns, Queensland, Australia; 10 Australian Institute of Tropical Health and Medicine, James Cook University, Townsville, Australia; 11 Department of Vascular and Endovascular Surgery, The Townsville Hospital, Townsville, Queensland, Australia; University of Illinois at Urbana-Champaign, UNITED STATES

## Abstract

**Objective:**

High plantar pressures are implicated in the development of diabetes-related foot ulcers. Whether plantar pressures remain high in patients with chronic diabetes-related foot ulcers over time is uncertain. The primary aim of this study was to compare plantar pressures at baseline and three and six months later in participants with chronic diabetes-related foot ulcers (cases) to participants without foot ulcers (controls).

**Methods:**

Standardised protocols were used to measure mean peak plantar pressure and pressure-time integral at 10 plantar foot sites (the hallux, toes, metatarsals 1 to 5, mid-foot, medial heel and lateral heel) during barefoot walking. Measurements were performed at three study visits: baseline, three and six months. Linear mixed effects random-intercept models were utilised to assess whether plantar pressures differed between cases and controls after adjusting for age, sex, body mass index, neuropathy status and follow-up time. Standardised mean differences (Cohen’s d) were used to measure effect size.

**Results:**

Twenty-one cases and 69 controls started the study and 16 cases and 63 controls completed the study. Cases had a higher mean peak plantar pressure at several foot sites including the toes (*p = 0*.*005*, Cohen’s d = 0.36) and mid-foot (*p = 0*.*01*, d = 0.36) and a higher pressure-time integral at the hallux (*p<0*.*001*, d = 0.42), metatarsal 1 (*p = 0*.*02*, d = 0.33) and mid-foot (*p = 0*.*04*, d = 0.64) compared to controls throughout follow-up. A reduction in pressure-time integral at multiple plantar sites over time was detected in all participants (*p<0*.*05*, *respectively)*.

**Conclusions:**

Plantar pressures assessed during gait are higher in diabetes patients with chronic foot ulcers than controls at several plantar sites throughout prolonged follow-up. Long term offloading is needed in diabetes patients with diabetes-related foot ulcers to facilitate ulcer healing.

## Introduction

Patients with diabetes-related foot ulcers (DFUs) have poor health-related quality of life and are at risk of prolonged hospitalisations [1–3]. DFUs frequently fail to heal and can remain stagnant in the inflammatory phase of healing increasing the risk of infection and limb amputation [4–8]. Therefore expediting the healing of DFUs is of paramount importance [1, 9, 10].

High plantar pressures have been implicated in the development of DFU by increasing the mechanical stress experienced by plantar tissue in the presence of diabetic peripheral neuropathy (DPN) [11–13]. Previous cross-sectional studies have reported that the pressures measured at specific plantar sites and resultant tissue stresses during gait are higher in people with active DFUs than controls [14–16]. Hence a frequent cause of delayed healing of DFUs is thought to be high plantar pressures during gait [14, 17, 18–19]. Studies have demonstrated much shorter DFU healing times when plantar stresses on ulcerated tissues were reduced by using offloading devices (such as total contact casts or removable cast walkers) [18]. It has also been suggested that patients with DFUs may adapt to these tissue stresses via the development of an alternative gait strategy to reduce plantar pressures [20, 21]. However, no longitudinal study has previously examined plantar pressures in patients with chronic DFUs [14, 19]. Therefore, measuring plantar pressures in people with DFUs during ulcer healing could provide importance guidance to pressure-offloading approaches with the aim of improving wound healing.

The aim of this study was to investigate plantar pressures at baseline and three and six months later in participants with DFUs (cases) compared to participants without DFUs (controls). We hypothesised that cases with DFUs would have significantly higher plantar pressures at baseline compared to controls and that these plantar pressure differences would remain during follow-up at three and six months.

## Methods

### Study design and setting

This was a longitudinal study which was nested in a case-control study. The study protocol and the baseline results of the study were previously published [14, 19]. All participants attended the Movement Analysis Laboratory, James Cook University, Townsville, Queensland, Australia on three separate occasions (baseline, first-follow-up at three months and second follow-up at six months) between July 2012 to November 2014.

### Participants

Twenty one participants with active unilateral plantar DFUs of more than 3 months duration (cases) and 69 type 2 diabetes mellitus participants without ulcers (controls) were initially recruited for this longitudinal study [19]. Inclusion criteria for the cases included adults (18 years or older) with a confirmed diagnosis of type 2 diabetes and a single active unilateral plantar DFU of longer than three months duration with an ankle-brachial pressure index (ABPI) greater than 0.8 in both limbs [19]. All patients with DFU had neuropathic DFUs. The control group comprised of adults with a confirmed diagnosis of type 2 diabetes without a history of DFUs and an ABPI greater than 0.8 in either limb [19]. The exclusion criteria were designed to avoid inclusion of participants with other conditions impacting on mobility or a condition that would likely mask the impact of a plantar foot ulcer on gait such as severe arthritis or a prior history of knee arthroplasty and have been detailed previously [19]. All participants were recruited from the Townsville Hospital and Health Service District, in Queensland, Australia between July 2012 and May 2014. The study was approved by two human research ethics committees (HREC): The Townsville Hospital HREC and the James Cook University HREC, (approval numbers HREC/12/QTHS/77 and H4693, respectively). Written informed consent was obtained from all participants [19].

### Participant characteristics

All anthropometric, haematological and clinical measurements were performed according to the study protocol [19]. Measures such as age, height, sex, ethnicity, monofilament sensation, the Michigan Neuropathy Symptom and Physical Assessment Scores and ABPIs were only assessed at baseline [14, 19, 20]. Each participant’s weight, body mass index (BMI), body fat percentage and waist and hip circumference, ulcer area, University of Texas Wound Classification Score (UTWCS) [19], glycated haemglobin A1c (HbA1c) and estimated glomerular filtration rate (eGFR) [19] were measured at each study-visit. We screened both cases and controls for the presence of DPN using several different methods following recognised guidelines [21, 22] and as detailed in our study protocol [19]. The screening consisted of using a 10g (size 5.07) monofilament sensation test at twenty plantar locations, a 128 Hz tuning fork sensation perception test and administration of the Michigan neuropathy screening instrument (MNSI) [19]. Where a participant was unable to detect the monofilament at five or less sites out of eight pre-defined sites, they were considered to have DPN [23]. Additionally, vibration sensation was assessed on a scale of 0 to 8 in each leg based on the number of times vibration commencement and cessation was felt accurately [24]. The same assessor (MEF) carried out all assessments. Good-to-excellent reproducibility (concordance correlation coefficients between 0.999 [95% Confidence Interval (CI): 0.999–0.999] and 0.998 [95% CI: 0.995–0.999]) were previously reported for all measurements performed in the study [25].

All participants received standard care between follow up visits external to their involvement in the study [18]. For cases this typically comprised of assessment and treatment of the ulcer by a podiatrist at least once every four weeks [18]. Most controls attended an annual foot-check with a podiatrist as suggested in National guidelines and had regular review of their diabetes control with a General Practitioner or Endocrinologist [18].

### Procedure used to measure plantar pressures

The Footscan^®^ pressure plate (RSscan International, Olen, Belgium) was used for plantar pressure assessment along with the associated Foot Scan ^®^ processing software. Plantar pressures were measured in both feet at baseline, three and six months follow-up visits. All cases with DFUs were given a standard single-layer generic film wound dressing to wear over the wound during gait examination to minimise the impact of wound dressings on the study results and to standardise the type of dressing during gait assessments and to minimise the risk of wound infection [19]. The dressings which were placed over the plantar foot ulcer remained intact for the entire duration of walking. In addition to the generic film adhesive, all dressings were further supported and reinforced using a single layer of hypoallergenic adhesive dressing tape. After data collection on the day of the visit, the ulcer site was cleaned with saline solution, disinfected and re-dressed with an appropriate foam dressing. The patient returned to their regular wound care appointment thereafter as per routine clinical care. The movement analysis laboratory floors were disinfected using a hospital grade disinfectant daily at the start and the end of the day. In addition, as a precautionary measure, participants with DFUs were only requested to weight bear while gait and plantar pressure assessments were being carried out and they were instructed to remain sitting with their feet off the ground as much as possible between assessments.

The validated three step approach for plantar pressure measurement was used [26]. A standard protocol for collecting plantar pressure data during gait was used with five assessments per participant for obtaining individual averages [19]. The pressure measurement software permitted masking of the foot to enable identification of plantar pressures at ten plantar sites in each foot. The sites included the plantar surfaces of the hallux, toes two to five, metatarsal one, metatarsal two, metatarsal three, metatarsal four, and metatarsal five, the mid-foot, the lateral heel and the medial heel. Mean peak plantar pressure (mpp) in N/cm^2^ and pressure-time integral (pti) in Ns/cm^2^ were the outcomes of interest for this study [19]. The mpp relates to the average peak pressure during a single step at a particular site. The pti is the area under the pressure-time curve and describes a pressure value for the total load exposure of a specific plantar site during a single step [27]. We previously reported that the coefficients of variation (CVs) were below 30% for 17 out of 20 mpp measurements, whereas the CVs were below 30% for 14 out of 20 pti measurements [25].

### Statistical analysis

The normality of continuous data was assessed using the Shapiro-Wilk test. Categorical data were reported as numbers and percentages (%) and continuous data were reported as means and standard deviations (SD), mean differences (Δ) or medians and interquartile ranges (IQRs) depending on the distribution of data. Characteristics of cases and controls were compared with Student’s t-test, Pearson's chi-square test or Fisher's exact test when assumptions for Chi-square tests were not met. We compared plantar pressures of ulcerated feet to plantar pressures averaged from the left and right feet of controls. Differences in mpp and pti over time were examined using linear mixed effects random-intercept models with individual participants as random effects and ulcer presence, months of follow-up, age, sex, body mass index (BMI) and the presence of neuropathy as fixed effects. The fixed effects were selected based on their previously established influence on plantar pressures [14, 20]. Within our statistical models, we assessed whether there was an association between ‘time’ and any changes in plantar pressure in all participants. We used an ‘interaction’ term within mixed effects models to assess whether any changes in plantar pressure over time differed between cases and controls. Where no significant interaction existed between ulcer presence and plantar pressures over time, the models were repeated excluding the interaction term.

Results of linear mixed effects models were reported using t-values, degrees of freedom (df) and p-values for estimated coefficients. Analysis of variance (ANOVA) was used to assess the overall goodness of fit of the linear mixed models for plantar pressure comparisons and changes over-time (see S1 and S2 Files). ANOVA results were reported in the main results as a measure of statistical significance. The statistical significance of outcomes was considered first by assessing the p-value obtained from ANOVA and then by assessing the p-value from the table of coefficients. When both p values were less than 0.05, a result was considered statistically significant. Standardised mean differences (Cohen’s d values) were calculated for all outcomes which were significantly different between groups using a previously published formula: standardised mean difference (d) = t (2/n) ^1/2^ [28, 29]. The size and direction of the difference was graded based on Cohen’s d as: <0.10 trivial difference; 0.10–0.20 small difference; 0.20–0.60 medium difference; 0.60–1.20 large difference and ≥1.20 a very large difference [30].

**Fig 1:**
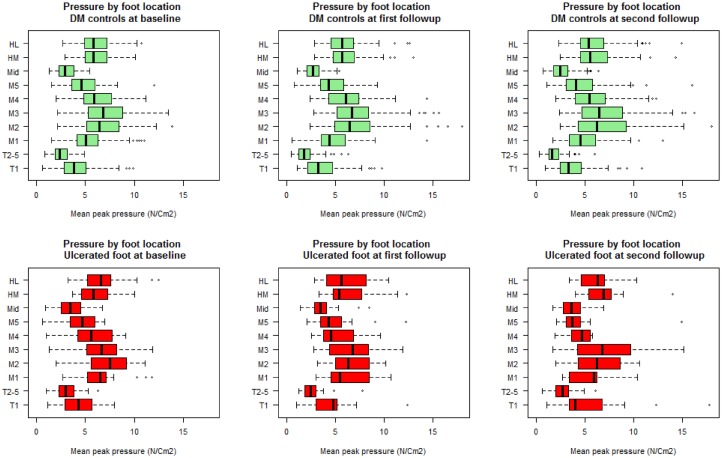
Site-specific mean peak pressures over time in participants with DFUs and participants without DFUs. Figure indicates the mean peak pressure at ten plantar sites in cases (red) and diabetes controls (DM controls) (green) at each visit. The x-axis has been scaled to allow for better data visualisation. All values are in N/Cm^2^ and are reported for 10 plantar foot sites. T1 = hallux (big-toe), T2-5 = toes two to five, M1 = metatarsal one, M2 = metatarsal two, M3 = metatarsal three, M4 = metatarsal four, M5 = metatarsal five, Mid = mid-foot, HM = medial heel and HL = lateral heel.

**Fig 2:**
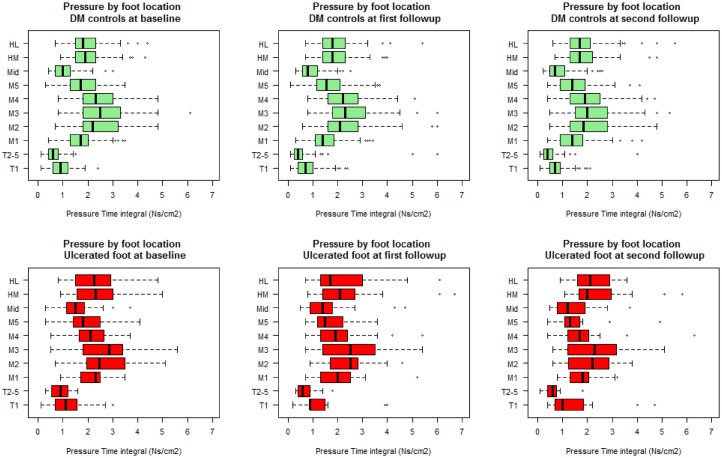
Site-specific pressure-time integrals over time in participants with DFUs and participants without DFUs. Figure indicates the pressure-time integral at ten plantar sites in cases (red) and diabetes controls (DM controls) (green) at each visit. The x-axis has been scaled to allow for better data visualisation. All values are in Ns/Cm^2^ and are reported for 10 plantar foot sites. T1 = hallux (big-toe), T2-5 = toes two to five, M1 = metatarsal one, M2 = metatarsal two, M3 = metatarsal three, M4 = metatarsal four, M5 = metatarsal five, Mid = mid-foot, HM = medial heel and HL = lateral heel.

In order to assess the impact of ulcer healing during follow-up we performed sensitivity analyses excluding participants with healed ulcers (see S3 File). These analyses showed similar results to those obtained by analysing all participants and therefore we have presented the latter results. SPSS 22.0 for Windows (SPSS Inc., Chicago, IL, USA) was used for the statistical comparisons of baseline demographic characteristics. The R (R Core Team, 2015) software was used for analysis of all longitudinal data with the ‘nlme’ package [31] for the mixed- effects models and for examining residual plots to check for deviations from homoscedasticity and normality assumptions. Summary plots of pti and mpp at the ten plantar sites were created for each time point (Figs 1 and 2).

## Results

### Recruitment and attrition of participants

Ninety participants commenced the study and were assessed at baseline (21 cases and 69 controls). Of those, five (24%) cases and six (9%) controls did not complete all follow-up visits. Prior to the three month follow-up visit [IQR 3–4 months], three cases (two due to orthopaedic surgery and one due to acute lower back pain) and two controls (one due to coronary artery bypass and one due to inability to attend) withdrew from the study. Prior to the six month follow-up visit [IQR 6–11.5 months], another three controls (one due to acute illness and two due to inability to attend and three cases (two due to hospitalisation and one due to inability to attend) also withdrew.

### Participant characteristics at baseline

The baseline data from this cohort were reported in an earlier manuscript [14]. Table 1 displays the baseline characteristics of the 21 cases and 69 controls that were initially recruited. There were no significant differences in age, sex, ethnicity, BMI, average HbA1c, smoking status or leg length between cases and controls at baseline. The presence of hammer-toe deformity was more common in cases at baseline (*p = 0*.*006*).

**Table 1:** Clinical and demographical characteristics of the enrolled study cohort at baseline.

Variable	Cases (n = 21)	Controls (n = 69)	*p-value*
Age at enrolment (years)	63.1 (± 10.6)	63.4 (± 9.6)	*0*.*905*
Males	15 (71.4%)	46 (66.7%)	*0*.*793*
Ethnicity			*1*.*000*
Caucasian	20 (95.2%)	65 (94.2%)	
Australian Aboriginal/Indigenous/Torres-strait Islander	1 (4.8%)	2 (2.9%)	
Other		2 (2.9%)	
Diabetes duration [years]^#^	16.6 (± 7.1)	10.7 (± 8.6)	*0*.*005*
HbA1c (mmol/l)^#^	58.9 (± 16.8)	54.8 (± 13.3)	*0*.*284*
Uses Insulin^#^	13 (61.9%)	19 (27.5%)	*0*.*005*
Smoking Status			*0*.*443*
Never Smoker	14 (66.7%)	34 (49.3%)	
Ex-Smoker	6 (28.6%)	29 (42.0%)	
Current Smoker	1 (4.8%)	6 (8.7%)	
History of hypertension	19 (90.5%)	46 (66.7%)	*0*.*049*
History of dyslipidaemia	14 (66.7%)	45 (65.2%)	*1*.*000*
History of stroke*	2 (9.5%)	2 (2.9%)	*0*.*231*
History of coronary heart disease	7 (33.3%)	18 (26.1%)	*0*.*581*
History of chronic heart failure	3 (14.3%)	9 (13.0%)	*1*.*000*
History of chronic pulmonary disease	4 (19.0%)	14 (20.3%)	*1*.*000*
History of chronic liver disease	2 (9.5%)	5 (7.2%)	*1*.*000*
History of chronic renal impairment	5 (23.8%)	10 (14.5%)	*0*.*506*
Height [cm]	173.7 (± 9.8)	169.6 (± 10.6)	*0*.*121*
Weight [kg]	102.5 (± 23.8)	91.3 (± 15.2)	*0*.*012*
BMI [Body Mass Index] [kg/m^2^]	34.0 (± 8.3)	31.8 (± 4.80)	*0*.*120*
Body Fact Percentage [% bf]	28.5 (± 13.7)	27.8 (± 12.6)	*0*.*834*
Waist Circumference [cm]	113.5 (± 17.9)	106.6 (± 11.2)	*0*.*035*
Hip Circumference[cm]	110.7 (± 18.9)	105.8 (± 10.2)	*0*.*120*
Left leg length [cm]	91.8 (± 7.1)	90.5 (± 5.6)	*0*.*390*
Right leg length [cm]	92.9 (± 8.0)	89.9 (± 11.4)	*0*.*266*
ABPI^	1.1 (± 0.2)	1.1 (± 0.2)	*0*.*913*
Monofilament score	7 (± 7)	18 (± 4)	*<0*.*001*
MNSI symptom score^#^	7 (± 1)	5 (± 2)	*<0*.*001*
MNSI physical assessment score^#^	7 (± 1)	2 (± 2)	*<0*.*001*
Foot-type			*0*.*166*
Pes planus foot type	14 (66.7%)	29 (42.0%)	
Normal arched foot type	4 (19.0%)	23 (33.3%)	
Pes cavus foot type	3 (14.3%)	17 (24.6%)	
First MTPJ RoM (degrees)	35.8 (± 14.4)	43.1 (± 15.1)	*0*.*052*
Ankle Joint RoM (restricted dorsiflexion)	17 (81.0%)	51 (73.9%)	*0*.*897*
Subtalar Joint RoM (restricted inversion/eversion)	2 (9.5%)	3 (4.4%)	*0*.*703*
Hallux Abducto Valgus deformity [32]			*0*.*955*
(No deformity)	14 (66.7%)	51 (73.9%)	
(Grade 1)	5 (23.8%)	13 (18.8%)	
(Grade 2)	1 (4.8%)	3 (4.3%)	
(Grade 3)	1 (4.8%)	2 (2.9%)	
Claw toe deformity	6 (28.6%)	11 (15.9%)	*0*.*213*
Hammer toe deformity	12 (57.1%)	16 (23.2%)	*0*.*006*
Mallet toe deformity	3 (14.3%)	14 (20.3%)	*0*.*752*

All data represents mean (± standard deviation) or number and percentages (%). Cases = foot ulcer group, controls = diabetes mellitus control group without ulcers. The reported test statistic indicates the t-statistic or Pearson’s Chi-square or Fisher’s exact test values with associated degrees of freedom. The reported p-values indicate main comparison outcomes from student’s t-tests, Pearson’s Chi squared tests or Fishers exact tests between groups. A significance level of p <0.05 was used throughout. Diabetes duration indicates fractions of years living with type 2 diabetes mellitus.

^ Ankle Brachial pressure Index = ABPI. ABPI values represented in the table are for ulcerated limbs of the Cases groups and the lowest reported in the control group. Monofilament score is out of a total of 20, measured at ten sites for each foot. MNSI scores indicate the total scores from the Michigan Neuropathy Screening Instrument in relation to the neuropathy symptom score and physical assessment score.

* Note that the four patients with stroke did not have a history of gait disturbance due to their stroke as the stroke only affected their speech function. Hallux Abducto Valgus (HAV) deformity grades were based on the Manchester scale as reported in the study protocol. RoM = Range of motion, restricted dorsiflexion incorporated people with < 10 degrees dorsiflexion.

### Participant characteristics during follow-up

Table 2 displays the anthropometric characteristics at baseline and the first and second follow-up visits for cases and controls. All cases had DFUs on the plantar aspect of the foot, including 16 ulcers (76.2%) under the fore-foot and five ulcers (23.8%) under the rear-foot. Most DFUs (81.0%) were superficial with a UTWCS grade of A1 or B1 [n = 17 (80.9%)] and the remainder extended to tendon or capsule [A2 = 3 (14.3%) and B2 = 1 (4.8%)]. Controls had a slightly longer follow-up period compared to cases (Table 2). There was a small decrease in the mean ulcer area at the first follow-up (Δ = 0.4 [SD = 0.8] mm^2^) and a slight increase in mean ulcer area at the second follow-up (Δ = 3.4 [3.6] mm^2^). The stance phase duration seemed to be longer in cases compared to controls throughout follow-up. Four (19.0%) DFUs healed during follow-up and remained healed (see Table 2). None of the controls developed DFUs during follow-up.

**Table 2:** Clinical and demographic characteristics of the study cohort at each follow-up.

Cohort at each follow-up						
Variable	Cases (n = 21)	Cases (n = 19)	Cases (n = 16)	Controls (n = 69)	Controls (n = 66)	Controls (n = 63)
	*Baseline*	*1*^*st*^ *follow-up*	*2*^*nd*^ *follow-up*	*Baseline*	*1*^*st*^ *follow-up*	*2*^*nd*^ *follow-up*
Number of months since baseline median [IQR]	-	3.0 [3.0–4.0]	6.0 [6.0–8.0]	-	4.0 [3.0–4.0]	9.0 [6.0–12.0]
Males (%)	15 (71.4%)	13 (68.4%)	12 (75.0%)	46 (66.7%)	45 (68.1%)	44 (69.8%)
Weight [kg] (SD)	102.6 (± 23.8)	106.9 (± 23.1)	108.5 (± 21.6)	91.3 (± 15.2)	91.0 (± 14.6)	91.4 (± 14.4)
BMI [Body Mass Index] [kg/m^2^] (SD)	34.0 (± 8.3)	35.3 (± 8.4)	35.6 (± 8.2)	31.8 (± 4.80)	31.4(± 4.7)	31.7 (± 4.8)
Body Fat Percentage [% bf] (SD)	28.5 (± 13.7)	27.5 (± 13.5)	26.0 (± 14.3)	27.8 (± 12.6)	27.5 (± 1.3)	29.6 (± 13.0)
Waist Circumference [cm] (SD)	113.5 (± 17.9)	106.0 (± 17.8)	109.0 (± 22.1)	106.6 (± 11.2)	105.0(± 10.0)	105.0 (± 10.2)
Hip Circumference[cm] (SD)	110.7 (± 18.9)	104.7 (± 9.7)	108.0 (± 11.3)	105.8 (± 10.2)	103.0 (± 9.3)	103.0 (± 9.3)
Stance phase duration (ms) (SD)	836 (± 115)	747 (± 99)	799 (± 137)	749 (± 93)	743 (± 57)	736 (± 56)
Ulcer grade (UTWCS) [33]						
A0	-	2 (10.5%)	4 (25.0%)			
A1	16 (76.2%)	14 (73.7%)	8 (50.0%)			
A2	3 (14.2%)	1 (5.3%)	1 (6.2%)			
A3	-	-	-			
B1	1 (4.7%)	2 (10.5%)	3 (18.8%)			
B2	1 (4.7%)	-	-			
Average ulcer area (mm^2^)*	20.3 (± 18.8)	19.9 (± 18.0)	23.3 (± 21.6)			

Data represents mean (± standard deviation; SD) or number and percentages (%), or median and [inter-quartile range; IQR]. Cases = foot ulcer group, controls = diabetes mellitus control group without foot ulcers. UTWCS = University of Texas Wound Classification Score. A0 = healed ulcer with complete epithelisation, A1 = superficial ulcer, A2 = ulcer which is down to the level of soft tissue, A3 = ulcer which is down to the level of bone, B1 = infected superficial ulcer, B2 = infected ulcer which is down to the level of soft tissue.

* Ulcer area was calculated excluding healed ulcers.

### Plantar pressure outcomes

#### Mean peak pressure

Cases had a significantly higher mpp at toes 2–5 (*p = 0*.*005*, d = 0.36) and the mid-foot (*p = 0*.*010*, d = 0.36) throughout follow-up compared to controls (see Table 3 and S1 File). Conversely, cases had a significantly lower mpp at metatarsal 4 compared to controls throughout follow-up (*p = 0*.*017*, d = -0.38) (see Table 3 and S1 File). Mpps decreased during follow-up at some sites including toes 2–5 (*p<0*.*001*, d = -0.38), metatarsal 1 (*p = 0*.*005*, d = -0.18) and the mid-foot (*p<0*.*001*, d = -0.36) in all participants (see Table 3 and Fig 1). The interaction term was insignificant between cases and controls.

**Table 3:** Mean peak pressure by group at baseline and during follow-up.

	Cases	Cases	Cases	Controls	Controls	Controls	ANOVA p-value for model Ulcer presence	t-value Ulcer presence (degrees of freedom [df])	p-value Ulcer presence	Cohen’s d Ulcer presence	ANOVA p-value for model Change over time	t-value Change over time (degrees of freedom [df])	p-value Change over time	Cohen’s d Change over time
*Mean peak pressure (mpp)N/cm*^*2*^	*Baseline*	*1*^*st*^*follow-up*	*2*^*nd*^ *follow-up*	*Baseline*	*1*^*st*^ *follow-up*	*2*^*nd*^ *follow-up*								
Toe 1/ Hallux	4.2 (± 1.9)	4.7 (± 2.7)	5.8 (± 4.4)	4.1 (± 1.8)	3.6 (± 1.8)	3.8 (± 1.8)	0.120	0.61 (86)	0.54	-	0.290	-1.40 (330)	0.160	-
Toes 2–5	3.1 (± 1.4)	2.8 (± 1.6)	2.9 (± 1.4)	2.5 (± 0.9)	1.9 (± 0.9)	1.9 (± 0.8)	0.005	2.37 (86)	0.02	0.36	<0.001	-4.88 (332)	<0.001	-0.38
Metatarsal 1	6.5 (± 2.4)	6.1 (± 2.4)	5.7 (± 2.5)	5.4 (± 1.9)	4.8 (± 1.9)	4.9 (± 2.1)	0.090	2.00 (86)	0.04	-	0.005	-2.28 (334)	0.020	-0.18
Metatarsal 2	7.2 (± 2.4)	6.7 (± 2.3)	6.4 (± 2.8)	6.9(± 2.4)	7.0 (± 3.1)	6.9 (± 3.1)	0.410	0.05 (86)	0.95	-	0.500	-0.28 (334)	0.770	-
Metatarsal 3	6.7 (± 2.5)	6.7 (± 2.7)	7.1 (± 3.7)	7.2 (± 2.6)	7.2 (± 2.7)	7.0 (± 3.1)	0.390	-1.12 (86)	0.26	-	0.650	-0.68 (333)	0.490	-
Metatarsal 4	5.6 (± 3.7)	5.3 (± 2.2)	5.3 (± 4.0)	6.2 (± 2.0)	6.1 (± 2.0)	5.7 (± 2.3)	0.017	-2.48 (86)	0.01	0.38	0.300	-1.36 (334)	0.170	-
Metatarsal 5	4.6 (± 1.7)	4.9 (± 2.6)	4.5 (± 3.0)	4.8 (± 1.6)	4.7 (± 1.7)	4.6 (± 2.2)	0.510	-1.22 (86)	0.22	-	0.640	-0.85 (330)	0.390	-
Mid Foot	3.7 (± 1.5)	3.9 (± 1.8)	3.7 (± 1.5)	3.1 (± 1.0)	2.9 (± 1.0)	2.6 (± 1.1)	0.010	2.35 (86)	0.02	0.36	<0.001	-4.59 (331)	<0.001	-0.36
Medial Heel	6.3(± 2.0)	6.5 (± 2.5)	6.9 (± 2.4)	6.1 (± 1.6)	6.0 (± 1.8)	6.0 (± 1.9)	0.630	0.16 (86)	0.87	-	0.590	0.60 (333)	0.540	-
Lateral Heel	6.8(± 2.5)	6.1 (± 2.5)	6.3 (± 2.1)	6.1 (± 1.7)	5.9 (± 1.8)	5.9 (± 2.1)	0.660	1.06 (86)	0.29	-	0.230	-0.73 (332)	0.460	-

All data represents mean peak pressures (mpp) and the reported values indicate the mean (± standard deviation) Cases = foot ulcer group, controls = diabetes mellitus control group without ulcers. - = not computed as this was not significantly different. The ANOVA p-value indicates values obtained an overall goodness of fit of statistical models and represent the overall significance of outcomes in the model. The p-values indicate the individual p-values obtained from the correlations tables from linear mixed effects models and the p-value for the effect of ulcer presence on plantar pressure and effect of time on plantar pressure.

#### Pressure-time integral

Cases had significantly higher ptis at the hallux (*p<0*.*001*, d = 0.42), plantar metatarsal 1 (*p = 0*.*02*, d = 0.33), the mid-foot (*p = 0*.*040*, d = 0.64), the medial heel (*p = 0*.020, d = 0.20) and the lateral heel (*p = 0*.*030*, d = 0.31) throughout follow-up compared to controls (see Table 4 and S2 File). The pti at the hallux (*p = 0*.*020*, d = -0.20), all metatarsals, including metatarsal 1 (*p<0*.*001*, d = -032) and the mid-foot (*p<0*.*001*, d = -0.33) decreased during follow-up in all participants (see Table 4 and Fig 2).

**Table 4:** Pressure-time integrals by group at baseline and during follow-up.

	Cases	Cases	Cases	Controls	Controls	Controls	ANOVA p-value for model Ulcer presence	t-value Ulcer presence (degrees of freedom [df])	p-value Ulcer presence	Cohen’s d Ulcer presence	ANOVA p-value for model Change over time	t-value Change over time (degrees of freedom [df])	p-value Change over time	Cohen’s d Change over time
*Pressure-time integral (pti) Ns/cm*^*2*^	*Baseline*	*1*^*st*^*follow-up*	*2*^*nd*^ *follow-up*	*Baseline*	*1*^*st*^ *follow-up*	*2*^*nd*^ *follow-up*								
Toe 1/ Hallux	1.2 (± 0.8)	1.4 (± 1.1)	1.5 (± 1.3)	0.9 (± 0.4)	0.8 (± 0.4)	0.8 (± 0.4)	<0.001	2.77 (86)	0.006	0.42	0.020	-2.54 (325)	0.011	-0.20
Toes 2–5	0.9 (± 0.4)	0.7 (± 0.4)	0.6 (± 0.4)	0.6 (± 0.3)	0.5 (± 0.8)	0.5 (± 0.8)	0.110	1.53 (86)	0.120	-	0.210	-0.96 (327)	0.330	-
Metatarsal 1	2.2 (± 0.7)	2.1 (± 1.1)	1.8 (± 0.8)	1.8 (± 0.7)	1.5 (± 0.7)	1.4 (± 0.7)	0.020	2.19 (86)	0.030	0.33	<0.001	-4.12 (328)	<0.001	-0.32
Metatarsal 2	2.7 (± 1.1)	2.4 (± 1.1)	2.1 (± 1.0)	2.5 (± 0.9)	2.3 (± 1.0)	2.1 (± 1.0)	0.690	0.87 (86)	0.380	-	<0.001	-3.41 (334)	0.007	-0.26
Metatarsal 3	2.7 (1.3)	2.6 (± 1.4)	2.4 (± 1.4)	2.6(± 1.0)	2.5 (± 1.0)	2.2 (± 1.0)	0.550	0.51 (86)	0.610	-	0.001	-3.68 (333)	<0.001	-0.29
Metatarsal 4	2.1 (± 0.9)	2.2 (± 1.3)	2.0 (± 1.4)	2.4 (± 0.9)	2.3 (± 0.8)	2.0 (± 0.9)	0.210	-1.53 (86)	0.120	-	0.004	-3.69 (333)	<0.001	-0.29
Metatarsal 5	1.9 (± 0.9)	1.8 (± 0.9)	1.6 (± 1.1)	1.8 (± 0.6)	1.7 (± 0.7)	1.5 (± 0.7)	0.970	-0.07 (86)	0.940	-	0.003	-2.84 (331)	0.004	-0.22
Mid Foot#	1.6 (± 0.9)	1.7 (± 1.2)	1.5 (± 0.9)	1.0 (± 0.4)	0.9 (± 0.4)	0.9 (± 0.5)	0.040	4.21 (86)	<0.001	0.64	<0.001	-4.26 (328)	<0.001	-0.33
Medial Heel	2.5 (± 1.2)	2.5(± 1.6)	2.5 (± 1.4)	2.0 (± 0.7)	1.9 (± 0.6)	1.8 (± 0.7)	0.020	1.24 (77)	0.210	0.20	0.180	-1.28 (77)	0.203	-
Lateral Heel	2.3 (± 1.1)	2.3 (± 1.5)	2.6 (± 2.1)	1.9 (± 0.7)	1.9 (± 0.6)	1.8 (± 0.8)	0.030	2.02 (86)	0.040	0.31	0.120	-1.37 (324)	0.171	-

All data represents pressure time integral (pti) and the reported values indicate the mean (± standard deviation) Cases = diabetic foot ulcer group, Controls = diabetes mellitus control group. - = not computed as this was not significantly different. The ANOVA p-value indicates values obtained an overall goodness of fit of statistical models and represent the overall significance of outcomes in the model. The p-values indicate the individual p-values obtained from the correlations tables from linear mixed effects models and the p-value for the effect of ulcer presence on plantar pressure and effect of time on plantar pressure.

^#^ = The interaction-term was significant for the DFU group for the mid-foot over-time; ANOVA p = 0.04, t = -2.05, df = 328, correlation p = 0.040, d = 0.16.

## Discussion

The main finding from this study was that plantar pressures (mpp and pti) were higher at multiple sites in cases with chronic DFUs compared to diabetes controls throughout a six month follow-up period. Overall, mpps and ptis at several sites significantly reduced over time in all participants. Although shorter wound healing times have been achieved by reducing plantar stresses on ulcerated tissue using offloading devices [18], whether plantar pressures actually remained elevated in people with chronic DFUs was largely unknown. The results from this study provide convincing evidence that plantar pressures remain elevated in people with DFUs and emphasise the need for long term pressure offloading in patients with chronic neuropathic DFUs to expedite and encourage ulcer healing, which is an important clinical consideration.

A number of longitudinal studies have previously assessed the association of plantar pressures with the subsequent risk of developing DFUs [34–37]. However, we are not aware of any longitudinal studies that have prospectively investigated plantar pressures in patients with existing active DFUs as reported here. Our findings suggest that patients with active DFUs have on-going higher plantar pressures at multiple sites by comparison to controls [14]. Boulton et al. (1987) reported that changes in the levels of plantar pressure may occur during a relatively short time in participants with DPN; however measurement repeatability needs to be considered in interpreting such data [35].

Our findings also suggest that overall plantar pressures reduced during repeated assessment. One possible reason for this is a familiarisation effect with repeated plantar pressure assessment. A previous study reported reductions in plantar pressure from baseline to follow-up for a majority of plantar sites in healthy participants [38]. This may indicate that as participants become more familiar with the walking environment and the plantar pressure protocol [19, 25], their plantar pressures show a relative reduction. These findings have some implications for researchers collecting plantar pressure data and may indicate that collecting plantar pressure data over several observations may lead to small gradual reductions in measurements. However, as the reductions in pressures occurred overall and as the sites identified to have the highest plantar pressures in cases compared to controls were similar throughout follow-up, whether there is added value in repeated plantar pressure measurements is uncertain [25, 39–41].

Our results support the need for sustained pressure offloading in patients with a history of chronic DFUs. Whether long term monitoring of plantar pressures can improve management of DFUs is controversial. Bus et al. (2011) suggested the use of in-shoe plantar pressure assessments to better inform pressure off-loading in participants with DPN at risk of developing DFU [42]. Despite this, a number of studies have suggested that plantar pressures are not routinely assessed in clinical practice [43, 44]. At present, very few centres around the world routinely utilise plantar pressures to ascertain levels of required offloading for patients at risk of DFUs [45, 46]. The high level of intra-participant, intra-device and inter-device variability of plantar pressure measurements is considered a deterrent to its routine use in clinical practice [25, 47]. This is a major limitation which needs further attention and improvement. Irrespective of this, plantar pressure measurements may indirectly assist in improving patient compliance with pressure offloading. One of the challenges for clinicians when communicating the importance of offloading with patients is the inability to demonstrate the need visually. The visualisation of plantar pressures using pressure measurements, in addition to quantitatively assessing the level of pressure, may provide an incentive to obtain better patient compliance with off-loading [48, 49].

A key area of future research focus in the field should be to characterise the important predictors of elevated plantar pressures in people with active DFUs. Recent work has outlined that foot-deformity and foot structure may be predictors of higher mid-foot plantar pressure in people with DPN and a history of DFUs [50]. Although we did not observe a statistical difference in the foot types between cases and controls, the higher mid-foot plantar pressures in cases may have been due to the presence of pes-planus foot-type. Other work has also identified that an increase in the viscoelasticity of plantar soft tissues, especially at the first metatarsophalangeal joint may be a crucial factor for elevated peak plantar pressures in people with DPN [51]. More recent work has outlined that new measures of plantar pressure such as the pressure gradient angle which quantifies the time-varying directions of plantar pressure may provide more valuable information regarding the plantar stressors experienced by people with DPN [52]. Whether measuring the viscoelasticity of plantar tissue and pressure gradient angles in people with active DFUs provides more insight on the biomechanical mechanisms underlying delayed wound healing is uncertain.

The limitations of our study include a small sample size in combination with a rather large number of statistical comparisons, the limited number of co-variates able to be used in statistical analyses and the inability to assess the association of plantar pressure and ulcer healing due to a small number of healed ulcers. It is also likely that reductions in plantar pressures that were observed were more representative of controls rather than cases as we had more controls than cases. The poor healing rate observed in our study (4 DFUs or 19% during six months follow-up) is representative of the inclusion criteria we used (i.e. people with DFUs of more than 3 months duration). Hence our results are representative of people with chronic DFUs and supports our earlier finding that following DFU healing, plantar pressures remain higher in people with a history of DFUs [53]. Therefore our results may not be applicable to people with DFUs of less than 3 months duration. A small increase in ulcer size at the second follow-up may have been due to cases with healing DFUs dropping out of the study, whereas cases with poorly healing DFUs remained. The length of follow-up varied between individuals and between the two groups; however, this was adjusted for in our analyses. A longer follow-up period may have provided further clarity on the relationship between elevated plantar pressures and ulcer healing, although this would have been limited by attrition.

As mentioned earlier, site specific mpp and pti have a variable level of reproducibility with repeated observations which may have also influenced our follow-up results [25]. We assessed barefoot plantar pressure rather than in-shoe pressure, as we wanted to investigate the foot-ground interaction in patients with DFUs without the influence of footwear. Our plantar pressure results seem to be lower than other values reported in the literature [16], but are consistent with other data obtained using the same pressure measurement system in participants with diabetes [54]. We assessed whether the reductions in plantar pressures over time were due to attrition of participants with higher plantar pressures at baseline, however excluding participants who were lost to follow-up did not influence the reductions of plantar pressure over time.

The strengths of this study include the longitudinal design, the reporting of reproducibility prior to data collection [25] and the use of statistical models to adjust plantar pressure outcomes for a number of key confounding factors including the presence of neuropathy. Our attrition rate was also significantly lower than the rate thought to be acceptable within the field. Our analyses accounted for numbers lost to follow-up and any differences in outcomes due to differences in follow-up times and the sample composition over time.

Our results highlight the importance of offloading in the long-term management of people with DFUs [18]. As the ideal percentage of plantar pressure reduction required to facilitate ulcer healing is yet to be determined [55], offloading efforts should aim to reduce plantar pressures as much as possible, using a 30% reduction recommended to prevent DFU development as a guide [45]. Given our results indicating that plantar pressures may show natural reductions during follow-up, it is imperative that clinicians should appreciate that natural changes in plantar pressures could occur with time. Future work should focus on how best to utilise plantar pressures in managing and preventing DFUs and in identifying alternate methods of reducing plantar pressure during gait [20].

## Conclusion

The findings from this study suggest that plantar pressures assessed during gait are higher in diabetes patients with chronic DFUs than controls throughout prolonged follow-up at several plantar sites. Long term offloading is needed in diabetes patients with chronic DFUs to facilitate ulcer healing.

## Supporting information

S1 File Outputs from linear mixed effects models of mean peak pressure (mpp). (DOCX)

S2 File Outputs from linear mixed effects models of pressure time integral (pti). (DOCX)

S3 File Outputs from linear mixed effects models of pressure-time integral (pti) and mean peak pressure (mpp) for active ulcer patients (excluding people with healed ulcers). (DOCX)

## Abbreviations

abpi: ankle brachial pressure index
ca: contact area
DFU: Diabetes foot ulcer
DPN-: Diabetic Peripheral Neuropathy
mpp: mean peak plantar pressure
msp: maximum sensor pressure
PAD: Peripheral Arterial Disease
pti: pressure-time integral
vgrf: vertical ground reaction force
