# Supplementary material for: Plantar pressures are elevated in people with longstanding diabetes-related foot ulcers during follow-up
Source: PLoS One. 2017 Aug 31;12(8):e0181916. doi: 10.1371/journal.pone.0181916 (PMC5578502; doi:10.1371/journal.pone.0181916)
Supplement: S1 File — (DOCX) [file pone.0181916.s001.docx]

**Supplementary File S1: Outputs from linear mixed effects models of mean peak pressure (mpp)**

1. Linear mixed-effects model of overall peak mpp

**With interaction term;**

numDF denDF F-value p-value

(Intercept) 1 336 2868.3033 <.0001

BMI 1 336 22.2077 <.0001

Age 1 86 0.0007 0.9783

Gender 1 86 0.0201 0.8876

hasNeuropathy 1 336 0.1454 0.7033

Months 1 336 3.2202 0.0736

UlcerPresence 1 86 0.1253 0.7242

Months:UlcerPresence 1 336 0.0956 0.7573

> summary(Overall_MPP.lme1)

Linear mixed-effects model fit by REML

Data: plantar

Subset: UlcerPresence != "Other foot"

AIC BIC logLik

1562.9 1603.35 -771.4501

Random effects:

Formula: ~1 | Participant

(Intercept) Residual

StdDev: 0.6659682 1.30569

Fixed effects: Average_pressure ~ BMI + Age + Gender + hasNeuropathy + Months + UlcerPresence + Months:UlcerPresence

Value Std.Error DF t-value p-value

(Intercept) 2.7387625 0.8751689 336 3.129410 0.0019

BMI 0.0777874 0.0180711 336 4.304509 0.0000

Age -0.0000240 0.0094150 86 -0.002545 0.9980

GenderFemale -0.0192555 0.2007307 86 -0.095927 0.9238

hasNeuropathyTRUE 0.0802336 0.2236492 336 0.358748 0.7200

Months -0.0301792 0.0169362 336 -1.781930 0.0757

UlcerPresenceThis foot 0.0381740 0.3240969 86 0.117786 0.9065

Months:UlcerPresenceThis foot 0.0171027 0.0553097 336 0.309216 0.7573

**Without interaction term;**

(Intercept) 1 337 2861.2580 <.0001

BMI 1 337 22.1855 <.0001

Age 1 86 0.0007 0.9787

Gender 1 86 0.0201 0.8877

hasNeuropathy 1 337 0.1486 0.7001

Months 1 337 3.2386 0.0728

UlcerPresence 1 86 0.1244 0.7252

> summary(Overall_MPP.lme1)

Linear mixed-effects model fit by REML

Data: plantar

Subset: UlcerPresence != "Other foot"

AIC BIC logLik

1557.042 1593.469 -769.5211

Random effects:

Formula: ~1 | Participant

(Intercept) Residual

StdDev: 0.6682983 1.303583

Fixed effects: Average_pressure ~ BMI + Age + Gender + hasNeuropathy + Months + UlcerPresence

Value Std.Error DF t-value p-value

(Intercept) 2.7094308 0.8717153 337 3.108160 0.0020

BMI 0.0787528 0.0178404 337 4.414284 0.0000

Age -0.0001815 0.0094112 86 -0.019287 0.9847

GenderFemale -0.0164422 0.2007903 86 -0.081887 0.9349

hasNeuropathyTRUE 0.0836343 0.2237043 337 0.373861 0.7087

Months -0.0285850 0.0160648 337 -1.779353 0.0761

UlcerPresenceThis foot 0.0944377 0.2677592 86 0.352697 0.7252

1. Linear mixed-effects model of mpp at plantar hallux (Toe 1)

**With interaction term;**

numDF denDF F-value p-value

(Intercept) 1 330 564.9056 <.0001

BMI 1 330 14.3284 0.0002

Age 1 86 0.9892 0.3227

Gender 1 86 1.1349 0.2897

hasNeuropathy 1 330 1.2167 0.2708

Months 1 330 1.0814 0.2992

UlcerPresence 1 86 2.3874 0.1260

Months:UlcerPresence 1 330 2.4713 0.1169

> summary(Toe1.lmeMPP3)

Linear mixed-effects model fit by REML

Data: plantar

Subset: UlcerPresence != "Other foot"

AIC BIC logLik

1694.461 1734.768 -837.2304

Random effects:

Formula: ~1 | Participant

(Intercept) Residual

StdDev: 1.452561 1.427228

Fixed effects: MeanPressure__T1A ~ BMI + Age + Gender + hasNeuropathy + Months + UlcerPresence + Months:UlcerPresence

Value Std.Error DF t-value p-value

(Intercept) 2.3871755 1.5290503 330 1.5612145 0.1194

BMI 0.0863942 0.0308097 330 2.8041261 0.0053

Age -0.0183286 0.0166860 86 -1.0984452 0.2751

GenderFemale -0.3043539 0.3573796 86 -0.8516262 0.3968

hasNeuropathyTRUE 0.3474723 0.3594907 330 0.9665682 0.3345

Months -0.0265858 0.0189797 330 -1.4007473 0.1622

UlcerPresenceThis foot 0.3092584 0.5039379 86 0.6136835 0.5410

Months:UlcerPresenceThis foot 0.1091259 0.0694170 330 1.5720346 0.1169

**Without interaction term;**

numDF denDF F-value p-value

(Intercept) 1 331 543.0710 <.0001

BMI 1 331 13.8867 0.0002

Age 1 86 0.9542 0.3314

Gender 1 86 1.0813 0.3013

hasNeuropathy 1 331 1.2682 0.2609

Months 1 331 1.1337 0.2878

UlcerPresence 1 86 2.2940 0.1335

> summary(Toe1.lmeMPP3)

Linear mixed-effects model fit by REML

Data: plantar

Subset: UlcerPresence != "Other foot"

AIC BIC logLik

1691.372 1727.669 -836.6858

Random effects:

Formula: ~1 | Participant

(Intercept) Residual

StdDev: 1.489356 1.423114

Fixed effects: MeanPressure__T1A ~ BMI + Age + Gender + hasNeuropathy + Months + UlcerPresence

Value Std.Error DF t-value p-value

(Intercept) 2.1638252 1.5520381 331 1.3941830 0.1642

BMI 0.0952149 0.0308471 331 3.0866703 0.0022

Age -0.0200882 0.0169836 86 -1.1828008 0.2401

GenderFemale -0.2788463 0.3643468 86 -0.7653319 0.4462

hasNeuropathyTRUE 0.3896937 0.3638086 331 1.0711502 0.2849

Months -0.0186940 0.0181858 331 -1.0279465 0.3047

UlcerPresenceThis foot 0.6837425 0.4514362 86 1.5145936 0.1335

1. Linear mixed-effects model of mpp at plantar toes 2-5

**With interaction term;**

numDF denDF F-value p-value

(Intercept) 1 332 871.5562 <.0001

BMI 1 332 18.1592 <.0001

Age 1 86 0.0024 0.9609

Gender 1 86 1.1143 0.2941

hasNeuropathy 1 332 1.8609 0.1734

Months 1 332 26.5041 <.0001

UlcerPresence 1 86 8.1877 0.0053

Months:UlcerPresence 1 332 0.0500 0.8233

> summary(Toe2to5.lmeMPP3)

Linear mixed-effects model fit by REML

Data: plantar

Subset: UlcerPresence != "Other foot"

AIC BIC logLik

1169.836 1210.19 -574.9178

Random effects:

Formula: ~1 | Participant

(Intercept) Residual

StdDev: 0.6265813 0.7838469

Fixed effects: MeanPressure__T2to5A ~ BMI + Age + Gender + hasNeuropathy + Months + UlcerPresence + Months:UlcerPresence

Value Std.Error DF t-value p-value

(Intercept) 0.9030310 0.7011639 332 1.287903 0.1987

BMI 0.0493433 0.0142786 332 3.455741 0.0006

Age -0.0020763 0.0075800 86 -0.273912 0.7848

GenderFemale -0.1041123 0.1623298 86 -0.641363 0.5230

hasNeuropathyTRUE 0.2164303 0.1712222 332 1.264032 0.2071

Months -0.0506551 0.0103645 332 -4.887377 0.0000

UlcerPresenceThis foot 0.5626485 0.2377030 86 2.367023 0.0202

Months:UlcerPresenceThis foot 0.0079114 0.0353977 332 0.223502 0.8233

**Without interaction term;**

numDF denDF F-value p-value

(Intercept) 1 333 868.3974 <.0001

BMI 1 333 18.1214 <.0001

Age 1 86 0.0025 0.9602

Gender 1 86 1.1094 0.2952

hasNeuropathy 1 333 1.8663 0.1728

Months 1 333 26.6304 <.0001

UlcerPresence 1 86 8.1564 0.0054

> summary(Toe2to5.lmeMPP3)

Linear mixed-effects model fit by REML

Data: plantar

Subset: UlcerPresence != "Other foot"

AIC BIC logLik

1163.038 1199.379 -572.5191

Random effects:

Formula: ~1 | Participant

(Intercept) Residual

StdDev: 0.6286227 0.7823682

Fixed effects: MeanPressure__T2to5A ~ BMI + Age + Gender + hasNeuropathy + Months + UlcerPresence

Value Std.Error DF t-value p-value

(Intercept) 0.8854029 0.6986954 333 1.267223 0.2060

BMI 0.0499470 0.0140741 333 3.548858 0.0004

Age -0.0021593 0.0075843 86 -0.284709 0.7766

GenderFemale -0.1025906 0.1625329 86 -0.631199 0.5296

hasNeuropathyTRUE 0.2183807 0.1713002 333 1.274842 0.2033

Months -0.0500031 0.0098759 333 -5.063172 0.0000

UlcerPresenceThis foot 0.5888883 0.2061973 86 2.855945 0.0054

1. Linear mixed-effects model of mpp at metatarsal 1

**With interaction term;**

numDF denDF F-value p-value

(Intercept) 1 334 1211.9876 <.0001

BMI 1 334 7.1210 0.0080

Age 1 86 1.1939 0.2776

Gender 1 86 1.3563 0.2474

hasNeuropathy 1 334 0.5819 0.4461

Months 1 334 7.9885 0.0050

UlcerPresence 1 86 2.8604 0.0944

Months:UlcerPresence 1 334 1.1310 0.2883

> summary(Met1.lmeMPP3)

Linear mixed-effects model fit by REML

Data: plantar

Subset: UlcerPresence != "Other foot"

AIC BIC logLik

1806.113 1846.516 -893.0567

Random effects:

Formula: ~1 | Participant

(Intercept) Residual

StdDev: 1.171974 1.695345

Fixed effects: MeanPressure__Met1A ~ BMI + Age + Gender + hasNeuropathy + Months + UlcerPresence + Months:UlcerPresence

Value Std.Error DF t-value p-value

(Intercept) 2.3407487 1.3684673 334 1.7104893 0.0881

BMI 0.0712249 0.0279171 334 2.5513015 0.0112

Age 0.0122858 0.0147612 86 0.8323031 0.4075

GenderFemale -0.2793138 0.3156398 86 -0.8849131 0.3787

hasNeuropathyTRUE 0.2451584 0.3403197 334 0.7203769 0.4718

Months -0.0507110 0.0222723 334 -2.2768654 0.0234

UlcerPresenceThis foot 0.9373576 0.4694581 86 1.9966799 0.0490

Months:UlcerPresenceThis foot -0.0775044 0.0728773 334 -1.0634912 0.2883

**Without interaction term;**

numDF denDF F-value p-value

(Intercept) 1 335 1228.9676 <.0001

BMI 1 335 7.1597 0.0078

Age 1 86 1.1986 0.2767

Gender 1 86 1.3739 0.2444

hasNeuropathy 1 335 0.5737 0.4493

Months 1 335 7.9427 0.0051

UlcerPresence 1 86 2.9128 0.0915

> summary(Met1.lmeMPP3)

Linear mixed-effects model fit by REML

Data: plantar

Subset: UlcerPresence != "Other foot"

AIC BIC logLik

1801.834 1838.218 -891.9172

Random effects:

Formula: ~1 | Participant

(Intercept) Residual

StdDev: 1.158968 1.698544

Fixed effects: MeanPressure__Met1A ~ BMI + Age + Gender + hasNeuropathy + Months + UlcerPresence

Value Std.Error DF t-value p-value

(Intercept) 2.4946401 1.3529614 335 1.8438369 0.0661

BMI 0.0662294 0.0273944 335 2.4176225 0.0162

Age 0.0129727 0.0146407 86 0.8860730 0.3780

GenderFemale -0.2925653 0.3131628 86 -0.9342274 0.3528

hasNeuropathyTRUE 0.2313675 0.3383524 335 0.6838064 0.4946

Months -0.0579698 0.0211979 335 -2.7346972 0.0066

UlcerPresenceThis foot 0.6885297 0.4034276 86 1.7066997 0.0915

1. Linear mixed-effects model of mpp at metatarsal 2

**With interaction term;**

numDF denDF F-value p-value

(Intercept) 1 334 1489.5555 <.0001

BMI 1 334 5.5299 0.0193

Age 1 86 1.4300 0.2351

Gender 1 86 0.2311 0.6319

hasNeuropathy 1 334 1.1048 0.2940

Months 1 334 0.4466 0.5044

UlcerPresence 1 86 0.6732 0.4142

Months:UlcerPresence 1 334 1.5276 0.2173

> summary(Met2.lmeMPP1)

Linear mixed-effects model fit by REML

Data: plantar

Subset: UlcerPresence != "Other foot"

AIC BIC logLik

2116.108 2156.51 -1048.054

Random effects:

Formula: ~1 | Participant

(Intercept) Residual

StdDev: 1.177797 2.571651

Fixed effects: MeanPressure__Met2A ~ BMI + Age + Gender + hasNeuropathy + Months + UlcerPresence + Months:UlcerPresence

Value Std.Error DF t-value p-value

(Intercept) 5.457016 1.6295022 334 3.348886 0.0009

BMI 0.082415 0.0337825 334 2.439580 0.0152

Age -0.018863 0.0175453 86 -1.075081 0.2853

GenderFemale -0.141673 0.3735301 86 -0.379281 0.7054

hasNeuropathyTRUE 0.473584 0.4193381 334 1.129361 0.2596

Months -0.009534 0.0334786 334 -0.284785 0.7760

UlcerPresenceThis foot 0.033364 0.6206617 86 0.053756 0.9573

Months:UlcerPresenceThis foot -0.134161 0.1085493 334 -1.235945 0.2173

**Without interaction term;**

numDF denDF F-value p-value

(Intercept) 1 335 1486.2526 <.0001

BMI 1 335 5.5189 0.0194

Age 1 86 1.4262 0.2357

Gender 1 86 0.2306 0.6323

hasNeuropathy 1 335 1.1046 0.2940

Months 1 335 0.4463 0.5046

UlcerPresence 1 86 0.6720 0.4146

> summary(Met2.lmeMPP1)

Linear mixed-effects model fit by REML

Data: plantar

Subset: UlcerPresence != "Other foot"

AIC BIC logLik

2113.032 2149.415 -1047.516

Random effects:

Formula: ~1 | Participant

(Intercept) Residual

StdDev: 1.179788 2.572971

Fixed effects: MeanPressure__Met2A ~ BMI + Age + Gender + hasNeuropathy + Months + UlcerPresence

Value Std.Error DF t-value p-value

(Intercept) 5.663641 1.6226417 335 3.490383 0.0005

BMI 0.075398 0.0333369 335 2.261694 0.0244

Age -0.017516 0.0175310 86 -0.999118 0.3205

GenderFemale -0.163556 0.3735250 86 -0.437871 0.6626

hasNeuropathyTRUE 0.454804 0.4194827 335 1.084202 0.2791

Months -0.022631 0.0317760 335 -0.712190 0.4768

UlcerPresenceThis foot -0.413797 0.5047977 86 -0.819727 0.4146

1. Linear mixed-effects model of mpp at metatarsal 3

**With interaction term;**

numDF denDF F-value p-value

(Intercept) 1 333 1234.8919 <.0001

BMI 1 333 2.5717 0.1097

Age 1 86 0.0231 0.8797

Gender 1 86 0.2316 0.6316

hasNeuropathy 1 333 0.0682 0.7942

Months 1 333 0.2005 0.6546

UlcerPresence 1 86 0.7258 0.3966

Months:UlcerPresence 1 333 0.5488 0.4594

> summary(Met3.lmeMPP3)

Linear mixed-effects model fit by REML

Data: plantar

Subset: UlcerPresence != "Other foot"

AIC BIC logLik

2066.662 2107.041 -1023.331

Random effects:

Formula: ~1 | Participant

(Intercept) Residual

StdDev: 1.517713 2.345442

Fixed effects: MeanPressure__Met3A ~ BMI + Age + Gender + hasNeuropathy + Months + UlcerPresence + Months:UlcerPresence

Value Std.Error DF t-value p-value

(Intercept) 5.224450 1.8140611 333 2.8799746 0.0042

BMI 0.056366 0.0370792 333 1.5201493 0.1294

Age 0.001026 0.0195876 86 0.0523545 0.9584

GenderFemale 0.157051 0.4184589 86 0.3753083 0.7084

hasNeuropathyTRUE 0.127228 0.4541045 333 0.2801744 0.7795

Months -0.021186 0.0308175 333 -0.6874525 0.4923

UlcerPresenceThis foot -0.717702 0.6430144 86 -1.1161530 0.2675

Months:UlcerPresenceThis foot 0.075953 0.1025313 333 0.7407767 0.4594

**Without interaction term;**

numDF denDF F-value p-value

(Intercept) 1 334 1237.5097 <.0001

BMI 1 334 2.5765 0.1094

Age 1 86 0.0231 0.8796

Gender 1 86 0.2322 0.6311

hasNeuropathy 1 334 0.0679 0.7946

Months 1 334 0.2004 0.6547

UlcerPresence 1 86 0.7272 0.3961

> summary(Met3.lmeMPP3)

Linear mixed-effects model fit by REML

Data: plantar

Subset: UlcerPresence != "Other foot"

AIC BIC logLik

2062.493 2098.856 -1022.247

Random effects:

Formula: ~1 | Participant

(Intercept) Residual

StdDev: 1.515615 2.344473

Fixed effects: MeanPressure__Met3A ~ BMI + Age + Gender + hasNeuropathy + Months + UlcerPresence

Value Std.Error DF t-value p-value

(Intercept) 5.098910 1.8042359 334 2.8260773 0.0050

BMI 0.060783 0.0365593 334 1.6625864 0.0973

Age 0.000187 0.0195344 86 0.0095548 0.9924

GenderFemale 0.173358 0.4174395 86 0.4152894 0.6790

hasNeuropathyTRUE 0.135826 0.4535180 334 0.2994934 0.7647

Months -0.014170 0.0293169 334 -0.4833361 0.6292

UlcerPresenceThis foot -0.462725 0.5426042 86 -0.8527864 0.3961

1. Linear mixed-effects model of mpp at metatarsal 4

**With interaction term;**

numDF denDF F-value p-value

(Intercept) 1 334 1881.3050 <.0001

BMI 1 334 16.4990 0.0001

Age 1 86 0.0001 0.9936

Gender 1 86 2.2793 0.1348

hasNeuropathy 1 334 0.0453 0.8316

Months 1 334 1.0547 0.3052

UlcerPresence 1 86 5.9250 0.0170

Months:UlcerPresence 1 334 0.7100 0.4000

> summary(Met4.lmeMPP3)

Linear mixed-effects model fit by REML

Data: plantar

Subset: UlcerPresence != "Other foot"

AIC BIC logLik

1857.581 1897.984 -918.7905

Random effects:

Formula: ~1 | Participant

(Intercept) Residual

StdDev: 0.9276584 1.875922

Fixed effects: MeanPressure__Met4A ~ BMI + Age + Gender + hasNeuropathy + Months + UlcerPresence + Months:UlcerPresence

Value Std.Error DF t-value p-value

(Intercept) 2.2592993 1.2385105 334 1.824207 0.0690

BMI 0.1059362 0.0255647 334 4.143854 0.0000

Age 0.0058660 0.0133365 86 0.439849 0.6612

GenderFemale 0.3514353 0.2837517 86 1.238531 0.2189

hasNeuropathyTRUE 0.0904918 0.3167861 334 0.285656 0.7753

Months -0.0332759 0.0244415 334 -1.361452 0.1743

UlcerPresenceThis foot -1.1455029 0.4618592 86 -2.480199 0.0151

Months:UlcerPresenceThis foot 0.0669121 0.0794088 334 0.842628 0.4000

**Without interaction term;**

numDF denDF F-value p-value

(Intercept) 1 335 1878.5428 <.0001

BMI 1 335 16.4816 0.0001

Age 1 86 0.0001 0.9934

Gender 1 86 2.2755 0.1351

hasNeuropathy 1 335 0.0459 0.8304

Months 1 335 1.0587 0.3043

UlcerPresence 1 86 5.9179 0.0171

> summary(Met4.lmeMPP3)

Linear mixed-effects model fit by REML

Data: plantar

Subset: UlcerPresence != "Other foot"

AIC BIC logLik

1853.062 1889.446 -917.5312

Random effects:

Formula: ~1 | Participant

(Intercept) Residual

StdDev: 0.9293498 1.874806

Fixed effects: MeanPressure__Met4A ~ BMI + Age + Gender + hasNeuropathy + Months + UlcerPresence

Value Std.Error DF t-value p-value

(Intercept) 2.1519825 1.2328397 335 1.745549 0.0818

BMI 0.1095238 0.0252243 335 4.341999 0.0000

Age 0.0052230 0.0133239 86 0.392005 0.6960

GenderFemale 0.3623380 0.2836608 86 1.277364 0.2049

hasNeuropathyTRUE 0.1005186 0.3167545 335 0.317339 0.7512

Months -0.0268236 0.0231888 335 -1.156750 0.2482

UlcerPresenceThis foot -0.9234779 0.3796141 86 -2.432676 0.0171

1. Linear mixed-effects model of mpp at metatarsal 5

**With interaction term;**

numDF denDF F-value p-value

(Intercept) 1 330 1424.1606 <.0001

BMI 1 330 4.9668 0.0265

Age 1 86 0.2735 0.6024

Gender 1 86 2.2852 0.1343

hasNeuropathy 1 330 0.0996 0.7525

Months 1 330 0.2138 0.6441

UlcerPresence 1 86 0.4214 0.5180

Months:UlcerPresence 1 330 1.4555 0.2285

> summary(Met5.lmeMPP3)

Linear mixed-effects model fit by REML

Data: plantar

Subset: UlcerPresence != "Other foot"

AIC BIC logLik

1751.443 1791.75 -865.7217

Random effects:

Formula: ~1 | Participant

(Intercept) Residual

StdDev: 0.8646562 1.677827

Fixed effects: MeanPressure__Met5A ~ BMI + Age + Gender + hasNeuropathy + Months + UlcerPresence + Months:UlcerPresence

Value Std.Error DF t-value p-value

(Intercept) 2.6673910 1.1386508 330 2.3425892 0.0197

BMI 0.0472290 0.0235477 330 2.0056763 0.0457

Age 0.0084953 0.0122117 86 0.6956637 0.4885

GenderFemale 0.3486994 0.2600000 86 1.3411513 0.1834

hasNeuropathyTRUE -0.1000295 0.2903300 330 -0.3445372 0.7307

Months -0.0185922 0.0219706 330 -0.8462322 0.3980

UlcerPresenceThis foot -0.5181319 0.4234937 86 -1.2234702 0.2245

Months:UlcerPresenceThis foot 0.0869452 0.0720667 330 1.2064549 0.2285

**Without interaction term;**

numDF denDF F-value p-value

(Intercept) 1 331 1426.8484 <.0001

BMI 1 331 4.9775 0.0263

Age 1 86 0.2744 0.6018

Gender 1 86 2.2880 0.1340

hasNeuropathy 1 331 0.1008 0.7510

Months 1 331 0.2120 0.6455

UlcerPresence 1 86 0.4223 0.5175

> summary(Met5.lmeMPP3)

Linear mixed-effects model fit by REML

Data: plantar

Subset: UlcerPresence != "Other foot"

AIC BIC logLik

1747.476 1783.774 -864.7379

Random effects:

Formula: ~1 | Participant

(Intercept) Residual

StdDev: 0.8625841 1.67934

Fixed effects: MeanPressure__Met5A ~ BMI + Age + Gender + hasNeuropathy + Months + UlcerPresence

Value Std.Error DF t-value p-value

(Intercept) 2.5159165 1.1307360 331 2.2250255 0.0268

BMI 0.0521053 0.0231829 331 2.2475729 0.0253

Age 0.0077540 0.0121850 86 0.6363515 0.5262

GenderFemale 0.3617895 0.2595195 86 1.3940747 0.1669

hasNeuropathyTRUE -0.0844109 0.2898270 331 -0.2912460 0.7710

Months -0.0103324 0.0209016 331 -0.4943346 0.6214

UlcerPresenceThis foot -0.2253900 0.3468337 86 -0.6498505 0.5175

1. Linear mixed-effects model of mpp at mid-foot

**With interaction term;**

numDF denDF F-value p-value

(Intercept) 1 331 1579.7929 <.0001

BMI 1 331 51.7785 <.0001

Age 1 86 2.5106 0.1167

Gender 1 86 0.0179 0.8939

hasNeuropathy 1 331 2.2618 0.1336

Months 1 331 25.8408 <.0001

UlcerPresence 1 86 5.6635 0.0195

Months:UlcerPresence 1 331 0.4803 0.4888

> summary(Midfoot.lmeMPP3)

Linear mixed-effects model fit by REML

Data: plantar

Subset: UlcerPresence != "Other foot"

AIC BIC logLik

1226.213 1266.544 -603.1066

Random effects:

Formula: ~1 | Participant

(Intercept) Residual

StdDev: 0.5907814 0.8593474

Fixed effects: MeanPressure__MidfootA ~ BMI + Age + Gender + hasNeuropathy + Months + UlcerPresence + Months:UlcerPresence

Value Std.Error DF t-value p-value

(Intercept) -0.6007410 0.6960472 331 -0.863075 0.3887

BMI 0.0957798 0.0143478 331 6.675561 0.0000

Age 0.0099127 0.0075003 86 1.321636 0.1898

GenderFemale 0.0394385 0.1598203 86 0.246768 0.8057

hasNeuropathyTRUE 0.2699260 0.1734904 331 1.555855 0.1207

Months -0.0518692 0.0112848 331 -4.596378 0.0000

UlcerPresenceThis foot 0.5895296 0.2503666 86 2.354666 0.0208

Months:UlcerPresenceThis foot -0.0280196 0.0404304 331 -0.693034 0.4888

**Without interaction term;**

numDF denDF F-value p-value

(Intercept) 1 332 1607.2943 <.0001

BMI 1 332 52.5412 <.0001

Age 1 86 2.5300 0.1154

Gender 1 86 0.0168 0.8971

hasNeuropathy 1 332 2.2326 0.1361

Months 1 332 25.7417 <.0001

UlcerPresence 1 86 5.7598 0.0186

> summary(Midfoot.lmeMPP3)

Linear mixed-effects model fit by REML

Data: plantar

Subset: UlcerPresence != "Other foot"

AIC BIC logLik

1220.103 1256.423 -601.0516

Random effects:

Formula: ~1 | Participant

(Intercept) Residual

StdDev: 0.5827409 0.8605277

Fixed effects: MeanPressure__MidfootA ~ BMI + Age + Gender + hasNeuropathy + Months + UlcerPresence

Value Std.Error DF t-value p-value

(Intercept) -0.5369643 0.6850904 332 -0.783786 0.4337

BMI 0.0936201 0.0139170 332 6.727032 0.0000

Age 0.0102088 0.0074180 86 1.376226 0.1723

GenderFemale 0.0344783 0.1582168 86 0.217918 0.8280

hasNeuropathyTRUE 0.2570018 0.1717621 332 1.496266 0.1355

Months -0.0540294 0.0108229 332 -4.992119 0.0000

UlcerPresenceThis foot 0.4920358 0.2050189 86 2.399954 0.0186

1. Linear mixed-effects model of mpp at medial heel

**With interaction term;**

numDF denDF F-value p-value

(Intercept) 1 333 2457.0872 <.0001

BMI 1 333 22.9266 <.0001

Age 1 86 0.1932 0.6614

Gender 1 86 0.0045 0.9468

hasNeuropathy 1 333 0.2063 0.6500

Months 1 333 0.2832 0.5949

UlcerPresence 1 86 0.2238 0.6374

Months:UlcerPresence 1 333 0.1479 0.7008

> summary(MHeel.lmeMPP3)

Linear mixed-effects model fit by REML

Data: plantar

Subset: UlcerPresence != "Other foot"

AIC BIC logLik

1704.347 1744.726 -842.1736

Random effects:

Formula: ~1 | Participant

(Intercept) Residual

StdDev: 0.9019723 1.541674

Fixed effects: MeanPressure__MHeelA ~ BMI + Age + Gender + hasNeuropathy + Months + UlcerPresence + Months:UlcerPresence

Value Std.Error DF t-value p-value

(Intercept) 2.5784554 1.1273614 333 2.287160 0.0228

BMI 0.1035397 0.0236513 333 4.377765 0.0000

Age 0.0044443 0.0121019 86 0.367238 0.7143

GenderFemale -0.0254642 0.2580941 86 -0.098662 0.9216

hasNeuropathyTRUE -0.1384218 0.2838415 333 -0.487673 0.6261

Months -0.0121619 0.0201262 333 -0.604280 0.5461

UlcerPresenceThis foot 0.0673358 0.4157760 86 0.161952 0.8717

Months:UlcerPresenceThis foot 0.0269281 0.0700146 333 0.384607 0.7008

**Without interaction term;**

numDF denDF F-value p-value

(Intercept) 1 334 2431.0468 <.0001

BMI 1 334 22.8248 <.0001

Age 1 86 0.1906 0.6635

Gender 1 86 0.0043 0.9481

hasNeuropathy 1 334 0.1928 0.6609

Months 1 334 0.2959 0.5869

UlcerPresence 1 86 0.2187 0.6412

> summary(MHeel.lmeMPP3)

Linear mixed-effects model fit by REML

Data: plantar

Subset: UlcerPresence != "Other foot"

AIC BIC logLik

1699.007 1735.369 -840.5033

Random effects:

Formula: ~1 | Participant

(Intercept) Residual

StdDev: 0.9112555 1.537736

Fixed effects: MeanPressure__MHeelA ~ BMI + Age + Gender + hasNeuropathy + Months + UlcerPresence

Value Std.Error DF t-value p-value

(Intercept) 2.5132978 1.1235867 334 2.236853 0.0260

BMI 0.1058421 0.0231479 334 4.572426 0.0000

Age 0.0040975 0.0121309 86 0.337775 0.7364

GenderFemale -0.0180014 0.2588948 86 -0.069532 0.9447

hasNeuropathyTRUE -0.1295641 0.2847014 334 -0.455088 0.6493

Months -0.0100799 0.0191834 334 -0.525450 0.5996

1. Linear mixed-effects model of mpp at lateral heel

**With interaction term;**

numDF denDF F-value p-value

(Intercept) 1 332 2147.2451 <.0001

BMI 1 332 14.2571 0.0002

Age 1 86 0.1301 0.7192

Gender 1 86 0.0316 0.8593

hasNeuropathy 1 332 0.3819 0.5370

Months 1 332 1.3924 0.2389

UlcerPresence 1 86 0.1903 0.6637

Months:UlcerPresence 1 332 1.5690 0.2112

> summary(LHeel.lmeMPP3)

Linear mixed-effects model fit by REML

Data: plantar

Subset: UlcerPresence != "Other foot"

AIC BIC logLik

1758.706 1799.061 -869.3531

Random effects:

Formula: ~1 | Participant

(Intercept) Residual

StdDev: 0.9461271 1.657672

Fixed effects: MeanPressure__LHeelA ~ BMI + Age + Gender + hasNeuropathy + Months + UlcerPresence + Months:UlcerPresence

Value Std.Error DF t-value p-value

(Intercept) 2.9375638 1.1926663 332 2.463023 0.0143

BMI 0.0953750 0.0249175 332 3.827623 0.0002

Age 0.0025060 0.0128145 86 0.195563 0.8454

GenderFemale -0.0445542 0.2729581 86 -0.163227 0.8707

hasNeuropathyTRUE -0.1785151 0.3008434 332 -0.593382 0.5533

Months -0.0157140 0.0216589 332 -0.725521 0.4686

UlcerPresenceThis foot 0.4563384 0.4324128 86 1.055330 0.2942

Months:UlcerPresenceThis foot -0.0922200 0.0736232 332 -1.252595 0.2112

**Without interaction term;**

numDF denDF F-value p-value

(Intercept) 1 333 2185.4072 <.0001

BMI 1 333 14.4227 0.0002

Age 1 86 0.1340 0.7152

Gender 1 86 0.0309 0.8608

hasNeuropathy 1 333 0.4071 0.5239

Months 1 333 1.3673 0.2431

UlcerPresence 1 86 0.1961 0.6590

> summary(LHeel.lmeMPP3)

Linear mixed-effects model fit by REML

Data: plantar

Subset: UlcerPresence != "Other foot"

AIC BIC logLik

1754.878 1791.219 -868.4392

Random effects:

Formula: ~1 | Participant

(Intercept) Residual

StdDev: 0.9304681 1.662547

Fixed effects: MeanPressure__LHeelA ~ BMI + Age + Gender + hasNeuropathy + Months + UlcerPresence

Value Std.Error DF t-value p-value

(Intercept) 3.1079630 1.1751428 333 2.644754 0.0086

BMI 0.0897591 0.0243440 333 3.687108 0.0003

Age 0.0033105 0.0126873 86 0.260928 0.7948

GenderFemale -0.0579129 0.2702992 86 -0.214255 0.8309

hasNeuropathyTRUE -0.1966338 0.2985429 333 -0.658645 0.5106

Months -0.0236877 0.0207055 333 -1.144030 0.2534

UlcerPresenceThis foot 0.1586217 0.3581929 86 0.442839 0.6590
