# Supplementary material for: Plantar pressures are elevated in people with longstanding diabetes-related foot ulcers during follow-up
Source: PLoS One. 2017 Aug 31;12(8):e0181916. doi: 10.1371/journal.pone.0181916 (PMC5578502; doi:10.1371/journal.pone.0181916)
Supplement: S2 File — (DOCX) [file pone.0181916.s002.docx]

**Supplementary File S2: Outputs from linear mixed effects models of pressure time integral (pti)**

1. Linear mixed-effects model of overall peak pti (average of ten sites)

**With interaction term;**

numDF denDF F-value p-value

(Intercept) 1 336 1711.6081 <.0001

BMI 1 336 24.4234 <.0001

Age 1 86 0.0238 0.8778

Gender 1 86 0.0288 0.8657

hasNeuropathy 1 336 0.0224 0.8811

Months 1 336 20.1784 <.0001

UlcerPresence 1 86 3.5575 0.0627

Months:UlcerPresence 1 336 0.0235 0.8782

> summary(Average_PTI.lme1)

Linear mixed-effects model fit by REML

Data: plantar

Subset: UlcerPresence != "Other foot"

AIC BIC logLik

672.4963 712.9463 -326.2481

Random effects:

Formula: ~1 | Participant

(Intercept) Residual

StdDev: 0.3317425 0.4319993

Fixed effects: Average_PTI ~ BMI + Age + Gender + hasNeuropathy + Months + UlcerPresence + Months:UlcerPresence

Value Std.Error DF t-value p-value

(Intercept) 0.8126380 0.3746451 336 2.169087 0.0308

BMI 0.0322488 0.0076079 336 4.238848 0.0000

Age -0.0014327 0.0040489 86 -0.353843 0.7243

GenderFemale 0.0006878 0.0866472 86 0.007939 0.9937

hasNeuropathyTRUE 0.0066154 0.0920214 336 0.071890 0.9427

Months -0.0240226 0.0056672 336 -4.238897 0.0000

UlcerPresenceThis foot 0.1985190 0.1253602 86 1.583589 0.1170

Months:UlcerPresenceThis foot 0.0028601 0.0186514 336 0.153347 0.8782

**Without interaction term;**

numDF denDF F-value p-value

(Intercept) 1 337 1709.7755 <.0001

BMI 1 337 24.4037 <.0001

Age 1 86 0.0239 0.8776

Gender 1 86 0.0287 0.8659

hasNeuropathy 1 337 0.0238 0.8775

Months 1 337 20.2493 <.0001

UlcerPresence 1 86 3.5556 0.0627

> summary(Average_PTI.lme1)

Linear mixed-effects model fit by REML

Data: plantar

Subset: UlcerPresence != "Other foot"

AIC BIC logLik

664.3923 700.8187 -323.1962

Random effects:

Formula: ~1 | Participant

(Intercept) Residual

StdDev: 0.3321926 0.4313205

Fixed effects: Average_PTI ~ BMI + Age + Gender + hasNeuropathy + Months + UlcerPresence

Value Std.Error DF t-value p-value

(Intercept) 0.8073751 0.3732237 337 2.163247 0.0312

BMI 0.0324257 0.0075200 337 4.311903 0.0000

Age -0.0014618 0.0040470 86 -0.361217 0.7188

GenderFemale 0.0012360 0.0866414 86 0.014266 0.9887

hasNeuropathyTRUE 0.0074063 0.0919998 337 0.080504 0.9359

Months -0.0237623 0.0053825 337 -4.414734 0.0000

UlcerPresenceThis foot 0.2077023 0.1101504 86 1.885625 0.0627

1. Linear mixed-effects model of pti at plantar hallux (Toe 1)

**With interaction term;**

numDF denDF F-value p-value

(Intercept) 1 325 391.9877 <.0001

BMI 1 325 3.8131 0.0517

Age 1 86 1.5193 0.2211

Gender 1 86 2.2964 0.1333

hasNeuropathy 1 325 0.5112 0.4751

Months 1 325 5.2327 0.0228

UlcerPresence 1 86 15.9866 0.0001

Months:UlcerPresence 1 325 2.7757 0.0967

> summary(Toe1.lme1)

Linear mixed-effects model fit by REML

Data: plantar

Subset: UlcerPresence != "Other foot"

AIC BIC logLik

648.842 689.0279 -314.421

Random effects:

Formula: ~1 | Participant

(Intercept) Residual

StdDev: 0.4041555 0.4114443

Fixed effects: PressureTimeIntegral__T1A ~ BMI + Age + Gender + hasNeuropathy + Months + UlcerPresence + Months:UlcerPresence

Value Std.Error DF t-value p-value

(Intercept) 1.2706834 0.4294190 325 2.9590757 0.0033

BMI 0.0047716 0.0086338 325 0.5526644 0.5809

Age -0.0080981 0.0046699 86 -1.7341191 0.0865

GenderFemale -0.1094994 0.1001935 86 -1.0928792 0.2775

hasNeuropathyTRUE 0.0490947 0.1014380 325 0.4839869 0.6287

Months -0.0140882 0.0055300 325 -2.5475933 0.0113

UlcerPresenceThis foot 0.3899064 0.1403127 86 2.7788390 0.0067

Months:UlcerPresenceThis foot 0.0320528 0.0192387 325 1.6660576 0.0967

**Without interaction term;**

numDF denDF F-value p-value

(Intercept) 1 326 375.9583 <.0001

BMI 1 326 3.6548 0.0568

Age 1 86 1.4784 0.2274

Gender 1 86 2.1899 0.1426

hasNeuropathy 1 326 0.5357 0.4647

Months 1 326 5.3641 0.0212

UlcerPresence 1 86 15.3541 0.0002

> summary(Toe1.lme5)

Linear mixed-effects model fit by REML

Data: plantar

Subset: UlcerPresence != "Other foot"

AIC BIC logLik

643.4829 679.6721 -312.7415

Random effects:

Formula: ~1 | Participant

(Intercept) Residual

StdDev: 0.4154676 0.4102423

Fixed effects: PressureTimeIntegral__T1A ~ BMI + Age + Gender + hasNeuropathy + Months + UlcerPresence

Value Std.Error DF t-value p-value

(Intercept) 1.2104900 0.4366348 326 2.772317 0.0059

BMI 0.0070972 0.0086817 326 0.817486 0.4142

Age -0.0085778 0.0047653 86 -1.800063 0.0754

GenderFemale -0.1008413 0.1023001 86 -0.985740 0.3270

hasNeuropathyTRUE 0.0584508 0.1028710 326 0.568196 0.5703

Months -0.0115408 0.0052759 326 -2.187469 0.0294

UlcerPresenceThis foot 0.4972745 0.1269066 86 3.918428 0.0002

Correlation:

(Intr) BMI Age GndrFm hNTRUE Months

BMI -0.735

Age -0.782 0.188

GenderFemale -0.049 -0.129 0.044

hasNeuropathyTRUE -0.069 -0.050 0.064 0.097

Months -0.023 -0.036 -0.006 0.007 -0.005

UlcerPresenceThis foot 0.186 -0.245 -0.145 0.105 -0.044 0.033

Standardized Within-Group Residuals:

Min Q1 Med Q3 Max

-5.5628704 -0.4802265 -0.1447698 0.3687730 5.3173350

Number of Observations: 419

Number of Groups: 90

1. Linear mixed-effects model of pti at plantar toes 2-5

**With interaction term;**

numDF denDF F-value p-value

(Intercept) 1 327 228.48523 <.0001

BMI 1 327 0.08686 0.7684

Age 1 86 0.09116 0.7634

Gender 1 86 0.02276 0.8804

hasNeuropathy 1 327 0.51566 0.4732

Months 1 327 1.51923 0.2186

UlcerPresence 1 86 2.57423 0.1123

Months:UlcerPresence 1 327 0.19296 0.6608

> summary(Toe2to5.lme1)

Linear mixed-effects model fit by REML

Data: plantar

Subset: UlcerPresence != "Other foot"

AIC BIC logLik

877.8546 918.089 -428.9273

Random effects:

Formula: ~1 | Participant

(Intercept) Residual

StdDev: 0.2476244 0.604608

Fixed effects: PressureTimeIntegral__T2to5A ~ BMI + Age + Gender + hasNeuropathy + Months + UlcerPresence + Months:UlcerPresence

Value Std.Error DF t-value p-value

(Intercept) 0.5944938 0.3661741 327 1.6235275 0.1054

BMI 0.0007991 0.0076111 327 0.1049913 0.9164

Age 0.0001197 0.0039224 86 0.0305257 0.9757

GenderFemale -0.0066221 0.0836365 86 -0.0791773 0.9371

hasNeuropathyTRUE -0.0735776 0.0941953 327 -0.7811171 0.4353

Months -0.0076548 0.0079245 327 -0.9659728 0.3348

UlcerPresenceThis foot 0.2245516 0.1463261 86 1.5345970 0.1286

Months:UlcerPresenceThis foot -0.0113829 0.0259129 327 -0.4392765 0.6608

**Without interaction term;**

numDF denDF F-value p-value

(Intercept) 1 328 230.20659 <.0001

BMI 1 328 0.08583 0.7697

Age 1 86 0.09209 0.7623

Gender 1 86 0.02293 0.8800

hasNeuropathy 1 328 0.52185 0.4706

Months 1 328 1.52300 0.2181

UlcerPresence 1 86 2.59376 0.1109

> summary(Toe2to5.lme2)

Linear mixed-effects model fit by REML

Data: plantar

Subset: UlcerPresence != "Other foot"

AIC BIC logLik

870.5769 906.8096 -426.2884

Random effects:

Formula: ~1 | Participant

(Intercept) Residual

StdDev: 0.2455026 0.6044487

Fixed effects: PressureTimeIntegral__T2to5A ~ BMI + Age + Gender + hasNeuropathy + Months + UlcerPresence

Value Std.Error DF t-value p-value

(Intercept) 0.6129779 0.3624328 328 1.6912873 0.0917

BMI 0.0001805 0.0074601 328 0.0241969 0.9807

Age 0.0002283 0.0038994 86 0.0585386 0.9535

GenderFemale -0.0085805 0.0831863 86 -0.1031481 0.9181

hasNeuropathyTRUE -0.0749942 0.0938149 328 -0.7993848 0.4246

Months -0.0087523 0.0075194 328 -1.1639558 0.2453

UlcerPresenceThis foot 0.1851026 0.1149337 86 1.6105167 0.1109

1. Linear mixed-effects model of pti at metatarsal 1

**With interaction term;**

(Intercept) 1 328 879.0398 <.0001

BMI 1 328 3.3174 0.0695

Age 1 86 0.9064 0.3437

Gender 1 86 0.9309 0.3373

hasNeuropathy 1 328 0.4755 0.4910

Months 1 328 20.5544 <.0001

UlcerPresence 1 86 5.6171 0.0200

Months:UlcerPresence 1 328 0.0521 0.8197

> summary(Met1.lme5)

Linear mixed-effects model fit by REML

Data: plantar

Subset: UlcerPresence != "Other foot"

AIC BIC logLik

892.4986 932.7573 -436.2493

Random effects:

Formula: ~1 | Participant

(Intercept) Residual

StdDev: 0.4552251 0.5672236

Fixed effects: PressureTimeIntegral__Met1A ~ BMI + Age + Gender + hasNeuropathy + Months + UlcerPresence + Months:UlcerPresence

Value Std.Error DF t-value p-value

(Intercept) 0.9905849 0.5127343 328 1.931965 0.0542

BMI 0.0164694 0.0105162 328 1.566098 0.1183

Age 0.0036058 0.0055258 86 0.652533 0.5158

GenderFemale -0.0770260 0.1179469 86 -0.653056 0.5155

hasNeuropathyTRUE 0.0719586 0.1241553 328 0.579585 0.5626

Months -0.0312050 0.0075397 328 -4.138755 0.0000

UlcerPresenceThis foot 0.3724794 0.1704003 86 2.185908 0.0315

Months:UlcerPresenceThis foot -0.0057318 0.0251213 328 -0.228166 0.8197

**Without interaction term;**

numDF denDF F-value p-value

(Intercept) 1 329 881.6556 <.0001

BMI 1 329 3.3271 0.0691

Age 1 86 0.9090 0.3431

Gender 1 86 0.9337 0.3366

hasNeuropathy 1 329 0.4763 0.4906

Months 1 329 20.5968 <.0001

UlcerPresence 1 86 5.6338 0.0198

> summary(Met1.lme5)

Linear mixed-effects model fit by REML

Data: plantar

Subset: UlcerPresence != "Other foot"

AIC BIC logLik

885.0194 921.2739 -433.5097

Random effects:

Formula: ~1 | Participant

(Intercept) Residual

StdDev: 0.4544753 0.5666248

Fixed effects: PressureTimeIntegral__Met1A ~ BMI + Age + Gender + hasNeuropathy + Months + UlcerPresence

Value Std.Error DF t-value p-value

(Intercept) 1.0030230 0.5091016 329 1.970183 0.0497

BMI 0.0160331 0.0103266 329 1.552596 0.1215

Age 0.0036728 0.0055097 86 0.666600 0.5068

GenderFemale -0.0781954 0.1176615 86 -0.664580 0.5081

hasNeuropathyTRUE 0.0713698 0.1239591 329 0.575753 0.5652

Months -0.0317271 0.0071737 329 -4.422668 0.0000

UlcerPresenceThis foot 0.3537344 0.1490310 86 2.373563 0.0198

1. Linear mixed-effects model of pti at metatarsal 2

**With interaction term;**

numDF denDF F-value p-value

(Intercept) 1 334 1013.5091 <.0001

BMI 1 334 5.0781 0.0249

Age 1 86 0.8761 0.3519

Gender 1 86 0.1139 0.7365

hasNeuropathy 1 334 0.9211 0.3379

Months 1 334 15.3208 0.0001

UlcerPresence 1 86 0.1526 0.6970

Months:UlcerPresence 1 334 1.0369 0.3093

> summary(Met2.lme2)

Linear mixed-effects model fit by REML

Data: plantar

Subset: UlcerPresence != "Other foot"

AIC BIC logLik

1183.043 1223.445 -581.5213

Random effects:

Formula: ~1 | Participant

(Intercept) Residual

StdDev: 0.5694828 0.8051635

Fixed effects: PressureTimeIntegral__Met2A ~ BMI + Age + Gender + hasNeuropathy + Months + UlcerPresence + Months:UlcerPresence

Value Std.Error DF t-value p-value

(Intercept) 1.8849873 0.6604620 334 2.854044 0.0046

BMI 0.0293585 0.0135362 334 2.168894 0.0308

Age -0.0067798 0.0071328 86 -0.950512 0.3445

GenderFemale -0.0218189 0.1526039 86 -0.142977 0.8866

hasNeuropathyTRUE 0.1678502 0.1641494 334 1.022545 0.3073

Months -0.0361025 0.0105793 334 -3.412561 0.0007

UlcerPresenceThis foot 0.1999138 0.2302194 86 0.868362 0.3876

Months:UlcerPresenceThis foot -0.0368926 0.0362301 334 -1.018286 0.3093

**Without interaction term;**

numDF denDF F-value p-value

(Intercept) 1 335 1023.4421 <.0001

BMI 1 335 5.1151 0.0244

Age 1 86 0.8857 0.3493

Gender 1 86 0.1151 0.7353

hasNeuropathy 1 335 0.9008 0.3433

Months 1 335 15.2782 0.0001

UlcerPresence 1 86 0.1532 0.6965

> summary(Met2.lme2)

Linear mixed-effects model fit by REML

Data: plantar

Subset: UlcerPresence != "Other foot"

AIC BIC logLik

1177.277 1213.661 -579.6385

Random effects:

Formula: ~1 | Participant

(Intercept) Residual

StdDev: 0.5651192 0.8061698

Fixed effects: PressureTimeIntegral__Met2A ~ BMI + Age + Gender + hasNeuropathy + Months + UlcerPresence

Value Std.Error DF t-value p-value

(Intercept) 1.9564368 0.6536894 335 2.992915 0.0030

BMI 0.0268313 0.0132495 335 2.025081 0.0437

Age -0.0063314 0.0070838 86 -0.893787 0.3739

GenderFemale -0.0307754 0.1516070 86 -0.202995 0.8396

hasNeuropathyTRUE 0.1573434 0.1633398 335 0.963289 0.3361

Months -0.0393063 0.0101042 335 -3.890079 0.0001

UlcerPresenceThis foot 0.0762943 0.1949233 86 0.391407 0.6965

1. Linear mixed-effects model of pti at metatarsal 3

**With interaction term;**

numDF denDF F-value p-value

(Intercept) 1 333 865.8440 <.0001

BMI 1 333 3.3268 0.0691

Age 1 86 0.7826 0.3788

Gender 1 86 0.0486 0.8260

hasNeuropathy 1 333 0.0021 0.9635

Months 1 333 14.9529 0.0001

UlcerPresence 1 86 0.3596 0.5503

Months:UlcerPresence 1 333 0.0009 0.9759

> summary(Met3.lme2)

Linear mixed-effects model fit by REML

Data: plantar

Subset: UlcerPresence != "Other foot"

AIC BIC logLik

1215.245 1255.623 -597.6224

Random effects:

Formula: ~1 | Participant

(Intercept) Residual

StdDev: 0.6920626 0.8179643

Fixed effects: PressureTimeIntegral__Met3A ~ BMI + Age + Gender + hasNeuropathy + Months + UlcerPresence + Months:UlcerPresence

Value Std.Error DF t-value p-value

(Intercept) 2.2878049 0.7612482 333 3.005334 0.0029

BMI 0.0234755 0.0154537 333 1.519088 0.1297

Age -0.0074750 0.0082524 86 -0.905790 0.3676

GenderFemale 0.0502162 0.1768473 86 0.283952 0.7771

hasNeuropathyTRUE 0.0089191 0.1847263 333 0.048283 0.9615

Months -0.0397742 0.0108088 333 -3.679791 0.0003

UlcerPresenceThis foot 0.1296866 0.2546181 86 0.509338 0.6118

Months:UlcerPresenceThis foot 0.0011273 0.0373430 333 0.030186 0.9759

**Without interaction term;**

numDF denDF F-value p-value

(Intercept) 1 333 865.8440 <.0001

BMI 1 333 3.3268 0.0691

Age 1 86 0.7826 0.3788

Gender 1 86 0.0486 0.8260

hasNeuropathy 1 333 0.0021 0.9635

Months 1 333 14.9529 0.0001

UlcerPresence 1 86 0.3596 0.5503

Months:UlcerPresence 1 333 0.0009 0.9759

> summary(Met3.lme2)

Linear mixed-effects model fit by REML

Data: plantar

Subset: UlcerPresence != "Other foot"

AIC BIC logLik

1215.245 1255.623 -597.6224

Random effects:

Formula: ~1 | Participant

(Intercept) Residual

StdDev: 0.6920626 0.8179643

Fixed effects: PressureTimeIntegral__Met3A ~ BMI + Age + Gender + hasNeuropathy + Months + UlcerPresence + Months:UlcerPresence

Value Std.Error DF t-value p-value

(Intercept) 2.2878049 0.7612482 333 3.005334 0.0029

BMI 0.0234755 0.0154537 333 1.519088 0.1297

Age -0.0074750 0.0082524 86 -0.905790 0.3676

GenderFemale 0.0502162 0.1768473 86 0.283952 0.7771

hasNeuropathyTRUE 0.0089191 0.1847263 333 0.048283 0.9615

Months -0.0397742 0.0108088 333 -3.679791 0.0003

UlcerPresenceThis foot 0.1296866 0.2546181 86 0.509338 0.6118

Months:UlcerPresenceThis foot 0.0011273 0.0373430 333 0.030186 0.9759

1. Linear mixed-effects model of pti at metatarsal 4

**With interaction term;**

(Intercept) 1 333 1229.1735 <.0001

BMI 1 333 12.9384 0.0004

Age 1 86 0.0875 0.7680

Gender 1 86 1.0657 0.3048

hasNeuropathy 1 333 0.0010 0.9750

Months 1 333 12.5699 0.0004

UlcerPresence 1 86 1.5902 0.2107

Months:UlcerPresence 1 333 0.7592 0.3842

> summary(Met4.lme3)

Linear mixed-effects model fit by REML

Data: plantar

Subset: UlcerPresence != "Other foot"

AIC BIC logLik

1143.981 1184.36 -561.9907

Random effects:

Formula: ~1 | Participant

(Intercept) Residual

StdDev: 0.4640009 0.7894899

Fixed effects: PressureTimeIntegral__Met4A ~ BMI + Age + Gender + hasNeuropathy + Months + UlcerPresence + Months:UlcerPresence

Value Std.Error DF t-value p-value

(Intercept) 0.9996352 0.5788980 333 1.726790 0.0851

BMI 0.0423375 0.0121423 333 3.486792 0.0006

Age 0.0000656 0.0062146 86 0.010563 0.9916

GenderFemale 0.1089505 0.1325431 86 0.822000 0.4133

hasNeuropathyTRUE 0.0088610 0.1456966 333 0.060818 0.9515

Months -0.0380220 0.0103080 333 -3.688593 0.0003

UlcerPresenceThis foot -0.3268367 0.2132498 86 -1.532647 0.1290

Months:UlcerPresenceThis foot 0.0312528 0.0358684 333 0.871318 0.3842

**Without interaction term;**

numDF denDF F-value p-value

(Intercept) 1 334 1208.2016 <.0001

BMI 1 334 12.7828 0.0004

Age 1 86 0.0892 0.7659

Gender 1 86 1.0515 0.3080

hasNeuropathy 1 334 0.0024 0.9609

Months 1 334 12.7217 0.0004

UlcerPresence 1 86 1.5503 0.2165

> summary(Met4.lme3)

Linear mixed-effects model fit by REML

Data: plantar

Subset: UlcerPresence != "Other foot"

AIC BIC logLik

1137.909 1174.272 -559.9547

Random effects:

Formula: ~1 | Participant

(Intercept) Residual

StdDev: 0.471155 0.7875667

Fixed effects: PressureTimeIntegral__Met4A ~ BMI + Age + Gender + hasNeuropathy + Months + UlcerPresence

Value Std.Error DF t-value p-value

(Intercept) 0.9341506 0.5787972 334 1.613952 0.1075

BMI 0.0447535 0.0119192 334 3.754740 0.0002

Age -0.0003735 0.0062495 86 -0.059761 0.9525

GenderFemale 0.1174901 0.1333890 86 0.880808 0.3809

hasNeuropathyTRUE 0.0173534 0.1465360 334 0.118424 0.9058

Months -0.0355001 0.0098283 334 -3.612012 0.0004

UlcerPresenceThis foot -0.2177822 0.1749085 86 -1.245121 0.2165

1. Linear mixed-effects model of pti at metatarsal 5

**With interaction term;**

(Intercept) 1 331 1078.9074 <.0001

BMI 1 331 14.4939 0.0002

Age 1 86 0.0173 0.8955

Gender 1 86 1.1446 0.2877

hasNeuropathy 1 331 0.0167 0.8971

Months 1 331 8.6463 0.0035

UlcerPresence 1 86 0.0011 0.9732

Months:UlcerPresence 1 331 0.0381 0.8454

> summary(Met5.lme3)

Linear mixed-effects model fit by REML

Data: plantar

Subset: UlcerPresence != "Other foot"

AIC BIC logLik

910.5715 950.9023 -445.2857

Random effects:

Formula: ~1 | Participant

(Intercept) Residual

StdDev: 0.3937828 0.5906332

Fixed effects: PressureTimeIntegral__Met5A ~ BMI + Age + Gender + hasNeuropathy + Months + UlcerPresence + Months:UlcerPresence

Value Std.Error DF t-value p-value

(Intercept) 0.5837968 0.4669151 331 1.250327 0.2121

BMI 0.0337305 0.0095378 331 3.536520 0.0005

Age 0.0010814 0.0050323 86 0.214895 0.8304

GenderFemale 0.1073754 0.1073859 86 0.999903 0.3202

hasNeuropathyTRUE -0.0156429 0.1163538 331 -0.134443 0.8931

Months -0.0222372 0.0078071 331 -2.848323 0.0047

UlcerPresenceThis foot -0.0114403 0.1613194 86 -0.070917 0.9436

Months:UlcerPresenceThis foot 0.0049489 0.0253614 331 0.195134 0.8454

**Without interaction term;**

numDF denDF F-value p-value

(Intercept) 1 332 1078.6879 <.0001

BMI 1 332 14.4925 0.0002

Age 1 86 0.0172 0.8960

Gender 1 86 1.1456 0.2875

hasNeuropathy 1 332 0.0164 0.8982

Months 1 332 8.6768 0.0035

UlcerPresence 1 86 0.0012 0.9730

> summary(Met5.lme3)

Linear mixed-effects model fit by REML

Data: plantar

Subset: UlcerPresence != "Other foot"

AIC BIC logLik

903.097 939.4163 -442.5485

Random effects:

Formula: ~1 | Participant

(Intercept) Residual

StdDev: 0.3941294 0.5897712

Fixed effects: PressureTimeIntegral__Met5A ~ BMI + Age + Gender + hasNeuropathy + Months + UlcerPresence

Value Std.Error DF t-value p-value

(Intercept) 0.5753065 0.4648021 332 1.237745 0.2167

BMI 0.0340192 0.0094184 332 3.611997 0.0004

Age 0.0010302 0.0050265 86 0.204946 0.8381

GenderFemale 0.1083195 0.1073088 86 1.009419 0.3156

hasNeuropathyTRUE -0.0148137 0.1162823 332 -0.127394 0.8987

Months -0.0217684 0.0074042 332 -2.939998 0.0035

UlcerPresenceThis foot 0.0047094 0.1385677 86 0.033986 0.9730

1. Linear mixed-effects model of pti at mid-foot

**With interaction term;**

(Intercept) 1 328 675.1698 <.0001

BMI 1 328 71.5620 <.0001

Age 1 86 1.9138 0.1701

Gender 1 86 0.1289 0.7204

hasNeuropathy 1 328 2.4313 0.1199

Months 1 328 27.4066 <.0001

UlcerPresence 1 86 13.5060 0.0004

Months:UlcerPresence 1 328 4.2264 0.0406

> summary(Midfoot.lme1)

Linear mixed-effects model fit by REML

Data: plantar

Subset: UlcerPresence != "Other foot"

AIC BIC logLik

552.9127 593.1714 -266.4564

Random effects:

Formula: ~1 | Participant

(Intercept) Residual

StdDev: 0.3541537 0.3655928

Fixed effects: PressureTimeIntegral__MidfootA ~ BMI + Age + Gender + hasNeuropathy + Months + UlcerPresence + Months:UlcerPresence

Value Std.Error DF t-value p-value

(Intercept) -1.0642895 0.3784549 328 -2.812196 0.0052

BMI 0.0589358 0.0076353 328 7.718857 0.0000

Age 0.0036861 0.0041067 86 0.897573 0.3719

GenderFemale 0.0148574 0.0878665 86 0.169090 0.8661

hasNeuropathyTRUE 0.1372038 0.0894516 328 1.533832 0.1260

Months -0.0207873 0.0048783 328 -4.261198 0.0000

UlcerPresenceThis foot 0.5163220 0.1227152 86 4.207482 0.0001

Months:UlcerPresenceThis foot -0.0344099 0.0167378 328 -2.055818 0.0406

**Without interaction term;**

numDF denDF F-value p-value

(Intercept) 1 329 711.1001 <.0001

BMI 1 329 75.2870 <.0001

Age 1 86 1.9839 0.1626

Gender 1 86 0.1285 0.7209

hasNeuropathy 1 329 2.2408 0.1354

Months 1 329 26.6847 <.0001

UlcerPresence 1 86 14.1280 0.0003

> summary(Midfoot.lme1)

Linear mixed-effects model fit by REML

Data: plantar

Subset: UlcerPresence != "Other foot"

AIC BIC logLik

548.6289 584.8834 -265.3145

Random effects:

Formula: ~1 | Participant

(Intercept) Residual

StdDev: 0.3411606 0.3698061

Fixed effects: PressureTimeIntegral__MidfootA ~ BMI + Age + Gender + hasNeuropathy + Months + UlcerPresence

Value Std.Error DF t-value p-value

(Intercept) -0.9890476 0.3672072 329 -2.693432 0.0074

BMI 0.0564461 0.0073590 329 7.670314 0.0000

Age 0.0040165 0.0039932 86 1.005829 0.3173

GenderFemale 0.0104229 0.0854578 86 0.121966 0.9032

hasNeuropathyTRUE 0.1213340 0.0878670 329 1.380882 0.1683

Months -0.0236249 0.0047088 329 -5.017129 0.0000

UlcerPresenceThis foot 0.4020595 0.1069670 86 3.758726 0.0003

1. Linear mixed-effects model of pti at medial heel

**With interaction term;**

numDF denDF F-value p-value

(Intercept) 1 77 145.85526 <.0001

BMI 1 77 10.64811 0.0016

Age 1 17 0.00695 0.9345

Gender 1 17 0.14825 0.7050

hasNeuropathy 1 17 0.00260 0.9599

Months 1 77 1.78679 0.1853

UlcerPresence 1 77 4.95035 0.0290

Months:UlcerPresence 1 77 0.20053 0.6555

> summary(MHeel.lme3)

Linear mixed-effects model fit by REML

Data: plantar

Subset: UlcerPresence != "DM Control"

AIC BIC logLik

313.009 338.4419 -146.5045

Random effects:

Formula: ~1 | Participant

(Intercept) Residual

StdDev: 0.7998315 0.7849555

Fixed effects: PressureTimeIntegral__MHeelA ~ BMI + Age + Gender + hasNeuropathy + Months + UlcerPresence + Months:UlcerPresence

Value Std.Error DF t-value p-value

(Intercept) -0.6813808 2.6341445 77 -0.2586725 0.7966

BMI 0.0816846 0.0373543 77 2.1867526 0.0318

Age 0.0016880 0.0261263 17 0.0646104 0.9492

GenderFemale 0.1698447 0.4768602 17 0.3561730 0.7261

hasNeuropathyTRUE 0.0077899 0.4465101 17 0.0174461 0.9863

Months -0.0423358 0.0329727 77 -1.2839672 0.2030

UlcerPresenceThis foot 0.2762475 0.2224673 77 1.2417441 0.2181

Months:UlcerPresenceThis foot 0.0192077 0.0428926 77 0.4478101 0.6555

**Without interaction term;**

numDF denDF F-value p-value

(Intercept) 1 77 145.85526 <.0001

BMI 1 77 10.64811 0.0016

Age 1 17 0.00695 0.9345

Gender 1 17 0.14825 0.7050

hasNeuropathy 1 17 0.00260 0.9599

Months 1 77 1.78679 0.1853

UlcerPresence 1 77 4.95035 0.0290

Months:UlcerPresence 1 77 0.20053 0.6555

> summary(MHeel.lme3)

Linear mixed-effects model fit by REML

Data: plantar

Subset: UlcerPresence != "DM Control"

AIC BIC logLik

313.009 338.4419 -146.5045

Random effects:

Formula: ~1 | Participant

(Intercept) Residual

StdDev: 0.7998315 0.7849555

Fixed effects: PressureTimeIntegral__MHeelA ~ BMI + Age + Gender + hasNeuropathy + Months + UlcerPresence + Months:UlcerPresence

Value Std.Error DF t-value p-value

(Intercept) -0.6813808 2.6341445 77 -0.2586725 0.7966

BMI 0.0816846 0.0373543 77 2.1867526 0.0318

Age 0.0016880 0.0261263 17 0.0646104 0.9492

GenderFemale 0.1698447 0.4768602 17 0.3561730 0.7261

hasNeuropathyTRUE 0.0077899 0.4465101 17 0.0174461 0.9863

Months -0.0423358 0.0329727 77 -1.2839672 0.2030

UlcerPresenceThis foot 0.2762475 0.2224673 77 1.2417441 0.2181

Months:UlcerPresenceThis foot 0.0192077 0.0428926 77 0.4478101 0.6555

1. Linear mixed-effects model of pti at lateral heel

**With interaction term;**

numDF denDF F-value p-value

(Intercept) 1 324 760.8715 <.0001

BMI 1 324 28.5010 <.0001

Age 1 86 0.0013 0.9708

Gender 1 86 0.0008 0.9768

hasNeuropathy 1 324 0.1777 0.6736

Months 1 324 2.4051 0.1219

UlcerPresence 1 86 4.8317 0.0306

Months:UlcerPresence 1 324 0.0294 0.8640

> summary(LHeel.lme3)

Linear mixed-effects model fit by REML

Data: plantar

Subset: UlcerPresence != "Other foot"

AIC BIC logLik

967.9961 1008.158 -473.998

Random effects:

Formula: ~1 | Participant

(Intercept) Residual

StdDev: 0.6144715 0.6052371

Fixed effects: PressureTimeIntegral__LHeelA ~ BMI + Age + Gender + hasNeuropathy + Months + UlcerPresence + Months:UlcerPresence

Value Std.Error DF t-value p-value

(Intercept) 0.1460835 0.6524799 324 0.223890 0.8230

BMI 0.0606785 0.0132365 324 4.584169 0.0000

Age -0.0022390 0.0070919 86 -0.315720 0.7530

GenderFemale 0.0341242 0.1517179 86 0.224919 0.8226

hasNeuropathyTRUE -0.0781691 0.1521704 324 -0.513695 0.6078

Months -0.0111907 0.0081691 324 -1.369887 0.1717

UlcerPresenceThis foot 0.4310275 0.2130023 86 2.023582 0.0461

Months:UlcerPresenceThis foot -0.0049758 0.0290345 324 -0.171375 0.8640

**Without interaction term;**

numDF denDF F-value p-value

(Intercept) 1 324 760.8715 <.0001

BMI 1 324 28.5010 <.0001

Age 1 86 0.0013 0.9708

Gender 1 86 0.0008 0.9768

hasNeuropathy 1 324 0.1777 0.6736

Months 1 324 2.4051 0.1219

UlcerPresence 1 86 4.8317 0.0306

Months:UlcerPresence 1 324 0.0294 0.8640

> summary(LHeel.lme3)

Linear mixed-effects model fit by REML

Data: plantar

Subset: UlcerPresence != "Other foot"

AIC BIC logLik

967.9961 1008.158 -473.998

Random effects:

Formula: ~1 | Participant

(Intercept) Residual

StdDev: 0.6144715 0.6052371

Fixed effects: PressureTimeIntegral__LHeelA ~ BMI + Age + Gender + hasNeuropathy + Months + UlcerPresence + Months:UlcerPresence

Value Std.Error DF t-value p-value

(Intercept) 0.1460835 0.6524799 324 0.223890 0.8230

BMI 0.0606785 0.0132365 324 4.584169 0.0000

Age -0.0022390 0.0070919 86 -0.315720 0.7530

GenderFemale 0.0341242 0.1517179 86 0.224919 0.8226

hasNeuropathyTRUE -0.0781691 0.1521704 324 -0.513695 0.6078

Months -0.0111907 0.0081691 324 -1.369887 0.1717

UlcerPresenceThis foot 0.4310275 0.2130023 86 2.023582 0.0461

Months:UlcerPresenceThis foot -0.0049758 0.0290345 324 -0.171375 0.8640
