# Supplementary material for: Plantar pressures are elevated in people with longstanding diabetes-related foot ulcers during follow-up
Source: PLoS One. 2017 Aug 31;12(8):e0181916. doi: 10.1371/journal.pone.0181916 (PMC5578502; doi:10.1371/journal.pone.0181916)
Supplement: S3 File — (DOCX) [file pone.0181916.s003.docx]

**Supplementary File S3: Outputs from linear mixed effects models of pressure-time integral (pti) and mean peak pressure (mpp) for active ulcer patients (excluding people with healed ulcers)**

1. Linear mixed-effects model of pti at plantar hallux (Toe 1)

numDF denDF F-value p-value

(Intercept) 1 368 526.6382 <.0001

BMI 1 368 3.2403 0.0727

Age 1 87 1.9807 0.1629

Gender 1 87 1.8843 0.1734

hasNeuropathy 1 368 0.3249 0.5690

Months 1 368 4.0814 0.0441

UlcerPresence 2 368 6.8004 0.0013

Months:UlcerPresence 2 368 1.4173 0.2437

> summary(Toe1.lme4)

Linear mixed-effects model fit by REML

Data: plantar

Subset: healed != "TRUE"

AIC BIC logLik

761.8699 811.3134 -368.9349

Random effects:

Formula: ~1 | Participant

(Intercept) Residual

StdDev: 0.3081626 0.4540367

Fixed effects: PressureTimeIntegral__T1A ~ BMI + Age + Gender + hasNeuropathy + Months + UlcerPresence + Months:UlcerPresence

Value Std.Error DF t-value p-value

(Intercept) 1.1430360 0.3570520 368 3.201315 0.0015

BMI 0.0058185 0.0070847 368 0.821276 0.4120

Age -0.0067367 0.0038436 87 -1.752679 0.0832

GenderFemale -0.0894264 0.0820691 87 -1.089648 0.2789

hasNeuropathyTRUE 0.0325220 0.0880313 368 0.369437 0.7120

Months -0.0128652 0.0060416 368 -2.129437 0.0339

UlcerPresenceOther foot 0.1164687 0.1259637 368 0.924621 0.3558

UlcerPresenceThis foot 0.2659722 0.1298228 368 2.048732 0.0412

Months:UlcerPresenceOther foot 0.0012368 0.0193401 368 0.063949 0.9490

Months:UlcerPresenceThis foot 0.0336794 0.0202940 368 1.659573 0.0979

1. Linear mixed-effects model of pti at the mid-foot

numDF denDF F-value p-value

(Intercept) 1 372 1126.7233 <.0001

BMI 1 372 89.2193 <.0001

Age 1 87 1.2090 0.2746

Gender 1 87 0.0188 0.8912

hasNeuropathy 1 372 0.3255 0.5686

Months 1 372 22.3185 <.0001

UlcerPresence 2 372 12.5377 <.0001

Months:UlcerPresence 2 372 1.3053 0.2723

> summary(Midfoot.lme4)

Linear mixed-effects model fit by REML

Data: plantar

Subset: healed != "TRUE"

AIC BIC logLik

701.3998 750.9484 -338.6999

Random effects:

Formula: ~1 | Participant

(Intercept) Residual

StdDev: 0.2301111 0.4349343

Fixed effects: PressureTimeIntegral__MidfootA ~ BMI + Age + Gender + hasNeuropathy + Months + UlcerPresence + Months:UlcerPresence

Value Std.Error DF t-value p-value

(Intercept) -0.6151879 0.29395531 372 -2.092794 0.0370

BMI 0.0500724 0.00588555 372 8.507683 0.0000

Age 0.0015122 0.00314570 87 0.480717 0.6319

GenderFemale 0.0118713 0.06679014 87 0.177740 0.8593

hasNeuropathyTRUE -0.0497466 0.07342815 372 -0.677486 0.4985

Months -0.0202610 0.00570874 372 -3.549120 0.0004

UlcerPresenceOther foot 0.2348929 0.10923283 372 2.150387 0.0322

UlcerPresenceThis foot 0.4705607 0.11360513 372 4.142072 0.0000

Months:UlcerPresenceOther foot -0.0293316 0.01815400 372 -1.615708 0.1070

Months:UlcerPresenceThis foot -0.0058366 0.01916758 372 -0.304503 0.7609

1. Linear mixed-effects model of pti at the medial heel

numDF denDF F-value p-value

(Intercept) 1 369 981.8962 <.0001

BMI 1 369 24.6310 <.0001

Age 1 87 0.1269 0.7226

Gender 1 87 0.0541 0.8167

hasNeuropathy 1 369 0.0303 0.8619

Months 1 369 6.1735 0.0134

UlcerPresence 2 369 5.5855 0.0041

Months:UlcerPresence 2 369 0.2112 0.8097

> summary(PressureTimeIntegral__MHeelA.lme4)

Linear mixed-effects model fit by REML

Data: plantar

Subset: healed != "TRUE"

AIC BIC logLik

1029.807 1079.277 -502.9035

Random effects:

Formula: ~1 | Participant

(Intercept) Residual

StdDev: 0.5400827 0.5825698

Fixed effects: PressureTimeIntegral__MHeelA ~ BMI + Age + Gender + hasNeuropathy + Months + UlcerPresence + Months:UlcerPresence

Value Std.Error DF t-value p-value

(Intercept) 0.3227259 0.5761459 369 0.560146 0.5757

BMI 0.0514386 0.0114088 369 4.508668 0.0000

Age 0.0003517 0.0062482 87 0.056292 0.9552

GenderFemale -0.0072982 0.1334755 87 -0.054678 0.9565

hasNeuropathyTRUE -0.0399027 0.1363566 369 -0.292635 0.7700

Months -0.0155352 0.0077824 369 -1.996198 0.0466

UlcerPresenceOther foot 0.1585537 0.1894964 369 0.836711 0.4033

UlcerPresenceThis foot 0.4449794 0.1907494 369 2.332796 0.0202

Months:UlcerPresenceOther foot -0.0158794 0.0254402 369 -0.624185 0.5329

Months:UlcerPresenceThis foot 0.0011520 0.0259636 369 0.044370 0.9646

1. Linear mixed-effects model of mpp at toes 2-5

numDF denDF F-value p-value

(Intercept) 1 372 1059.4529 <.0001

BMI 1 372 16.5599 0.0001

Age 1 87 0.0003 0.9857

Gender 1 87 1.6580 0.2013

hasNeuropathy 1 372 0.7723 0.3801

Months 1 372 24.9706 <.0001

UlcerPresence 2 372 4.3677 0.0133

Months:UlcerPresence 2 372 0.3132 0.7313

> summary(MeanPressure__T2to5A.lme4)

Linear mixed-effects model fit by REML

Data: plantar

Subset: healed != "TRUE"

AIC BIC logLik

1300.89 1350.438 -638.4449

Random effects:

Formula: ~1 | Participant

(Intercept) Residual

StdDev: 0.5579372 0.8104035

Fixed effects: MeanPressure__T2to5A ~ BMI + Age + Gender + hasNeuropathy + Months + UlcerPresence + Months:UlcerPresence

Value Std.Error DF t-value p-value

(Intercept) 1.2421174 0.6420807 372 1.934519 0.0538

BMI 0.0414545 0.0127820 372 3.243197 0.0013

Age -0.0030946 0.0069212 87 -0.447125 0.6559

GenderFemale -0.1312129 0.1478539 87 -0.887450 0.3773

hasNeuropathyTRUE 0.1188120 0.1583136 372 0.750485 0.4534

Months -0.0501655 0.0106745 372 -4.699570 0.0000

UlcerPresenceOther foot 0.4584259 0.2315946 372 1.979432 0.0485

UlcerPresenceThis foot 0.4665668 0.2316619 372 2.013998 0.0447

Months:UlcerPresenceOther foot 0.0278555 0.0352728 372 0.789714 0.4302

Months:UlcerPresenceThis foot 0.0038419 0.0360258 372 0.106643 0.9151

1. Linear mixed-effects model of mpp at metatarsal 1

numDF denDF F-value p-value

(Intercept) 1 376 1347.4568 <.0001

BMI 1 376 2.4325 0.1197

Age 1 87 0.8970 0.3462

Gender 1 87 2.0705 0.1538

hasNeuropathy 1 376 0.0826 0.7740

Months 1 376 11.6050 0.0007

UlcerPresence 2 376 1.7986 0.1669

Months:UlcerPresence 2 376 2.5803 0.0771

> summary(MeanPressure__Met1A.lme4)

Linear mixed-effects model fit by REML

Data: plantar

Subset: healed != "TRUE"

AIC BIC logLik

2024.626 2074.279 -1000.313

Random effects:

Formula: ~1 | Participant

(Intercept) Residual

StdDev: 1.08026 1.777707

Fixed effects: MeanPressure__Met1A ~ BMI + Age + Gender + hasNeuropathy + Months + UlcerPresence + Months:UlcerPresence

Value Std.Error DF t-value p-value

(Intercept) 3.195991 1.2957085 376 2.4665972 0.0141

BMI 0.052060 0.0258320 376 2.0153191 0.0446

Age 0.009247 0.0139476 87 0.6629988 0.5091

GenderFemale -0.367657 0.2970983 87 -1.2374929 0.2192

hasNeuropathyTRUE 0.101079 0.3231809 376 0.3127632 0.7546

Months -0.050525 0.0232672 376 -2.1714946 0.0305

UlcerPresenceOther foot 0.579219 0.4741215 376 1.2216671 0.2226

UlcerPresenceThis foot 0.877121 0.4718005 376 1.8590920 0.0638

Months:UlcerPresenceOther foot -0.163167 0.0755898 376 -2.1585888 0.0315

Months:UlcerPresenceThis foot -0.085053 0.0766948 376 -1.1089749 0.2681

1. Linear mixed-effects model of mpp at mid-foot

numDF denDF F-value p-value

(Intercept) 1 374 2142.2526 <.0001

BMI 1 374 50.9655 <.0001

Age 1 87 1.3041 0.2566

Gender 1 87 0.1087 0.7424

hasNeuropathy 1 374 0.0205 0.8863

Months 1 374 26.7127 <.0001

UlcerPresence 2 374 4.6814 0.0098

Months:UlcerPresence 2 374 2.1780 0.1147

> summary(MidfootA.lme4)

Linear mixed-effects model fit by REML

Data: plantar

Subset: healed != "TRUE"

AIC BIC logLik

1415.695 1465.296 -695.8477

Random effects:

Formula: ~1 | Participant

(Intercept) Residual

StdDev: 0.4432027 0.9536539

Fixed effects: MeanPressure__MidfootA ~ BMI + Age + Gender + hasNeuropathy + Months + UlcerPresence + Months:UlcerPresence

Value Std.Error DF t-value p-value

(Intercept) 0.0795491 0.5998975 374 0.132604 0.8946

BMI 0.0844479 0.0120688 374 6.997210 0.0000

Age 0.0052826 0.0064214 87 0.822648 0.4130

GenderFemale 0.0701915 0.1361534 87 0.515532 0.6075

hasNeuropathyTRUE -0.0060273 0.1515855 374 -0.039762 0.9683

Months -0.0510075 0.0123787 374 -4.120602 0.0000

UlcerPresenceOther foot 0.2728499 0.2352180 374 1.159987 0.2468

UlcerPresenceThis foot 0.5220658 0.2461407 374 2.121005 0.0346

Months:UlcerPresenceOther foot -0.0838971 0.0408025 374 -2.056179 0.0405

Months:UlcerPresenceThis foot -0.0037631 0.0431827 374 -0.087143 0.9306
